# Supplementary material for: Performance and patients’ satisfaction with the A7+TouchCare insulin patch pump system: A randomized controlled non-inferiority study
Source: PLoS One. 2023 Aug 24;18(8):e0289684. doi: 10.1371/journal.pone.0289684 (PMC10449223; doi:10.1371/journal.pone.0289684)
Supplement: S1 Table — (DOCX) [file pone.0289684.s001.docx]

**Tab****le S1: Skin tolerability and adverse events between inclusion and Visit 3 (Safety Population)**

|  | **Omnipod N=45** | **A7+ TouchCare N=48** | **P-value** |
| --- | --- | --- | --- |
| Irritation/itching | 12 (26.7%) | 13 (27.1%) | 0.964 |
| Redness | 11 (24.4%) | 18 (37.5%) | 0.174 |
| Other adverse events | 4 (8.9%) | 15 (31.3%) | 0.010 |

Data are number and % of patients; Chi² test or Fisher exact test
