# Supplementary material for: Performance and patients’ satisfaction with the A7+TouchCare insulin patch pump system: A randomized controlled non-inferiority study
Source: PLoS One. 2023 Aug 24;18(8):e0289684. doi: 10.1371/journal.pone.0289684 (PMC10449223; doi:10.1371/journal.pone.0289684)
Supplement: S1 File — (PDF) [file pone.0289684.s003.pdf]

Try the modernized ClinicalTrials.gov beta website. Learn more about the modernization effort.

MEDTRUM A7+ TouchCare Insulin Patch Pump (MedInPS) (MedInPS)

The safety and scientific validity of this study is the responsibility of the study sponsor and investigators. Listing a study does not mean it has been evaluated by the U.S. Federal Government. Read our disclaimer for details.

ClinicalTrials.gov Identifier: NCT04223973

Recruitment Status : Completed
First Posted : January 13, 2020
Last Update Posted : June 11, 2021

Sponsor:
Medtrum France

Information provided by (Responsible Party):
Medtrum France

Tracking Information

**First Submitted Date** ICMJE
December 11, 2019
**First Posted Date** ICMJE
January 13, 2020

Last Update Posted Date

June 11, 2021

Actual Study Start Date ICMJE

January 29, 2020

Actual Primary Completion Date

March 1, 2021 (Final data collection date for primary outcome measure)

Current Primary Outcome Measures ICMJE
(submitted: January 7, 2020)

Estimation of A1C based on average blood glucose measured by continuous glucose sensor [Time Frame: 3 months]

A1C will be estimated by a Continuous Glucose Measurement (FreeStyle Libre®) for all patients, at baseline and end of the study. The main objective is to demonstrate Non-inferiority efficacy (PP then ITT) of Medtrum A7+ insulin patch pump vs Insulet Omnipod® insulin patch pump (2 randomized groups). For the primary outcome, A1C will be estimated and appear in percentage based on assessment done by a Continuous Glucose Monitoring system. The A1C obtained in real life for the group using the comparator pump (Omnipod®) is estimated at 7,8%. Assuming that blood glucose level will be the same with Medtrum pump and based on a defined non-inferiority margin Δ = + 0,4 (following FDA Guidance for Industry Diabetes Mellitus: Developing Drugs and Therapeutic Biologics for Treatment and Prevention).

Original Primary Outcome Measures ICMJE

Same as current

Change History

Complete list of historical versions of study NCT04223973 on ClinicalTrials.gov Archive Site

Current Secondary Outcome Measures ICMJE
(submitted: January 12, 2020)

- Report Patients' physiological A1C difference -as measured at the laboratory-between the active group (Medtrum Pump) vs Control Group (comparator device) in order to assess non inferiority [Time Frame: 3 months]

  A1C is expressed in percentage based on Laboratory measurement. A1C is obtained & measured from a blood sample.
- Compare The Time spent in Range for the active group (Medtrum Pump) vs Control Group Patients (comparator device) in order to assess non inferiority [Time Frame: 3 months]

  Glycemic Time In Range is the Time spent in the target. It is expressed in % of time. Time in Range is automatically calculated from the Sensor data wear.

- Compare Patients satisfaction for the active group (Medtrum Pump) vs Control Group Patients (comparator device) in order to assess non inferiority [Time Frame: 3 months]

  Number of patients satisfied is expressed in percentage. Satisfaction to be measured by a Patient Self- questionnaire through a Visual Scoring scale ranking from 1 (the less) to 5 (the most).
- Compare number of pump dysfonction for the active group (Medtrum Pump) vs Control Group Patients (comparator device) in order to assess non inferiority [Time Frame: 3 months]

  (1) Number of technical issues related to the devices uses (reported by patient)
- Compare pump tolerance for the active group (Medtrum Pump) vs Control Group Patients (comparator device) in order to assess non inferiority [Time Frame: 3 months]

  1. Number of incidents related to tolerance of the devices (reported by patient)
  2. Severity of incidents related to tolerance of the devices (reported by patient)

Original Secondary Outcome Measures ICMJE
(submitted: January 7, 2020)

- Compare Laboratory A1C of the active group (Medtrum Pump) vs Control Group Patients (comparator device) in order to assess non inferiority [Time Frame: 3 months]

  A1c is expressed in percentage. Laboratory measurement obtained from a blood sample. 2 measurements: before visit 1 and before the last visit.
- Compare The Time spent in Range for the active group (Medtrum Pump) vs Control Group Patients (comparator device) in order to assess non inferiority [Time Frame: 3 months]

  Glycemic Time In Range is the Time spent in the target. It is expressed in % of time. Time in Range is automatically calculated from the Sensor data wear.
- Compare Patients satisfaction for the active group (Medtrum Pump) vs Control Group Patients (comparator device) in order to assess non inferiority [Time Frame: 3 months]

  Number of patients satisfied is expressed in percentage. Satisfaction to be measured by a Patient Self- questionnaire through a Visual Scoring scale ranking from 1 (the less) to 5 (the most).
- Compare number of pump dysfonction for the active group (Medtrum Pump) vs Control Group Patients (comparator device) in order to assess non inferiority [Time Frame: 3 months]

  (1) Number of technical issues related to the devices uses (reported by patient)
- Compare pump tolerance for the active group (Medtrum Pump) vs Control Group Patients (comparator device) in order to assess non inferiority [Time Frame: 3 months]

  1. Number of incidents related to tolerance of the devices (reported by patient)
  2. Severity of incidents related to tolerance of the devices (reported by patient)

Current Other Pre-specified Outcome Measures

Not Provided

Original Other Pre-specified Outcome Measures

Not Provided

Descriptive Information

Brief Title ICMJE

MEDTRUM A7+ TouchCare Insulin Patch Pump (MedInPS)

Official Title ICMJE

Demonstration Study of the Interest of the MEDTRUM A7+ TouchCare Insulin Patch Pump Versus INSULET Omnipod® Patch Pump

Brief Summary

Demonstration study of the interest of the MEDTRUM A7+ TouchCare insulin patch pump versus INSULET Omnipod® patch pump

Detailed Description

Compare the daily use of two single usage patch pumps and demonstrate the interest of use of the A7+TouchCare® (Medtrum) patch pump for type 1 & 2 diabetes management.

Main objective is an estimation of Patients A1C based on the average blood glucose (obtained from a Flash glucose monitoring) and to compare it in both groups.

Secondary objectives:

- comparison of mean A1C (laboratory values) between the two groups at Baseline and study end. Comparison to be established in a non-inferiority
- Observe Skin & overall Tolerance, Technical incidents with the device (failure, disconnection, catheter obstruction…)
- Colllection of Glucose metrics (minimum, maximum, average/median, standard deviation, out of range value, percentage of time in range, glucose variability)

Study Type ICMJE

Interventional

Study Phase ICMJE

Not Applicable

Study Design ICMJE

Allocation: Randomized
Intervention Model: Parallel Assignment
Intervention Model Description:

> Randomized controlled trial (RCT), open-label with two arms, multicentric national, prospective, non-inferiority methodology versus reimbursed comparator.

Masking: None (Open Label)
Primary Purpose: Treatment

Condition ICMJE

Diabetes Mellitus

Intervention ICMJE

- Device: Medtrum A7+ insulin Pump

  Use of the Medtrum pump during 3 months in combination with a CGM
- Biological: Lab A1C

  Measure of Lab A1C at baseline and end of study

Study Arms ICMJE

- Experimental: Active Group

  Using the Medtrum Pump A7+ during 3 months

  Interventions:
  - Device: Medtrum A7+ insulin Pump
  - Biological: Lab A1C
- Active Comparator: Control Group

  using the usual Insulet Patch pump

  Intervention: Biological: Lab A1C

Publications *

Not Provided

*** Includes publications given by the data provider as well as publications identified by ClinicalTrials.gov Identifier (NCT Number) in Medline.**

Recruitment Information

Recruitment Status ICMJE

Completed

Actual Enrollment ICMJE
(submitted: August 11, 2020)

82

Original Estimated Enrollment ICMJE
(submitted: January 7, 2020)

75

Actual Study Completion Date ICMJE

June 1, 2021

Actual Primary Completion Date

March 1, 2021 (Final data collection date for primary outcome measure)

Eligibility Criteria ICMJE

Inclusion Criteria:

- Patient with type 1 or 2 diabetes, 18 years of age and over
- Patients already equipped with Omnipod® ® (INSULET) insulin patch pump and Abbott FreeStyleLibre sensor®.
- A1C ranking from : >= 6,5% to <= 9.5%
- Any type of rapid insulin except FIASP (which can be substituted if necessary) with 60 UI max per day (unauthorized use of insulin supplements by pen injector)
- Patient able to receive and understand study information, give written informed consent, and easily participate to the trial
- Patient affiliated to the French social security system

Exclusion Criteria:

- - Patient already participating in another study
- Patient under the protection of justice or under guardianship or curatorship
- Type 2 diabetic patient requiring a daily insulin dose > 60 IU
- Patients not suitable for suing insulin pump such as: severe psychiatric disorders, rapidly progressing ischemic or proliferative retinopathy before laser treatment, and exposure to high magnetic fields
- Patients allergic to nickel and adhesive
- Pregnant women, breastfeeding women
- Or any other criteria as appreciated by the study investigator.

Sex/Gender ICMJE

Sexes Eligible for Study:
All

Ages ICMJE

18 Years and older (Adult, Older Adult)

Accepts Healthy Volunteers ICMJE

No

Contacts ICMJE

Contact information is only displayed when the study is recruiting subjects

Listed Location Countries ICMJE

France

Removed Location Countries

Administrative Information

NCT Number ICMJE

NCT04223973

Other Study ID Numbers ICMJE

2019-A02566-51

Has Data Monitoring Committee

Yes

U.S. FDA-regulated Product

Studies a U.S. FDA-regulated Drug Product:
No

Studies a U.S. FDA-regulated Device Product:
No

IPD Sharing Statement ICMJE

Plan to Share IPD:
No

Current Responsible Party

Medtrum France

Original Responsible Party

Same as current

Current Study Sponsor ICMJE

Medtrum France

Original Study Sponsor ICMJE

Same as current

**Collaborators** ICMJE	*Not Provided*
**Investigators** ICMJE	**Principal Investigator:**<br>Freddy PENFORNIS<br>Centre Hospitalier Francilien, Corbeil
**PRS Account**	Medtrum France
**Verification Date**	June 2021

ICMJE **Data element required by the International Committee of Medical Journal Editors and the World Health Organization ICTRP**
