# Supplementary material for: Performance and patients’ satisfaction with the A7+TouchCare insulin patch pump system: A randomized controlled non-inferiority study
Source: PLoS One. 2023 Aug 24;18(8):e0289684. doi: 10.1371/journal.pone.0289684 (PMC10449223; doi:10.1371/journal.pone.0289684)
Supplement: S2 File — (PDF) [file pone.0289684.s004.pdf]

EVALUATION DE L'INTERET DE LA POMPE PATCH MEDTRUM A7+ TOUCHCARE® VERSUS LA POMPE PATCH INSULET OMNIPOD®

Protocole d'étude interventionnelle

Version finale 1.3 du 13/05/2020

N° ID-RCB : 2019-A02566-51
Référence promoteur : MedInPS

Promoteur de l'étude

MEDTRUM France
88 T Avenue du Général Leclerc,
Boulogne Billancourt, France

Directeur Commercial

Mélanie Marmounier
88 T Avenue du Général Leclerc,
Boulogne Billancourt

Investigateur Principal

Pr Alfred Penfornis
Service d'Endocrinologie, Diabétologie et Maladies métaboliques
Centre Hospitalier Sud-Francilien de Corbeil-Essonnes (91)

CRO prestataire

Axonal-Biostatem
215 av. Georges Clemenceau
92024 Nanterre cedex
Email

CONFIDENTIEL
« Ce document est la propriété de MEDTRUM et ne peut – en tout ou partie – être transmis, reproduit, publié ou utilisé sans son autorisation expresse. »

PAGE DE VALIDATION DU PROTOCOLE

Evaluation de l'intérêt de la pompe patch Medtrum A7+ TouchCare® versus la pompe patch Insulet Omnipod®

Protocole version finale 1.3 du 13/05/2020 (ID-RCB 2019-A02566-51)

Représentant du Promoteur :

**Mélanie Marmounier**<br>Directeur Commercial et Réglementaire<br>MEDTRUM France<br>88 Avenue du Général Leclerc, Boulogne Billancourt<br>Tél : 06 20 59 12 24	Signature :<br>[redacted]

Investigateur principal de l'étude :

**Pr Alfred Penfornis**<br>Service d'Endocrinologie, Diabétologie et Maladies métaboliques<br>Centre Hospitalier Sud-Francilien de Corbeil-Essonnes (91)	Signature :<br>[redacted]

Logistique de l'étude :

**AXONAL-BIOSTATEM**<br>Le Clemenceau 2, 215 Avenue Georges Clemenceau<br>92000 Nanterre	
**Julien le Dall**<br>Chef de projet<br>jledall@axonal.com	Signature :<br>[redacted]

COMITÉ SCIENTIFIQUE

Pour cette étude clinique, MEDTRUM, le Promoteur, a choisi un Comité Scientifique constitué d'experts diabétologues prescrivant très régulièrement à leurs patients des pompes à insuline. Ce Comité Scientifique sera chargé de valider la pertinence de cette étude : objectifs, méthodologie et qualité scientifique du projet. Il apportera son soutien dans la conception de l'étude, contribuera à la rédaction du protocole, organisera des réunions et rédigera des publications de l'étude.

Membres du Comité Scientifique :

Pr Alfred Penfornis (Coordinateur de l'étude)
Service d'Endocrinologie, Diabétologie et Maladies métaboliques
Centre Hospitalier Sud-Francilien de Corbeil-Essonnes (91)
alfred.penfornis@chsf.fr
Tél : 01 61 69 30 86

Dr Jennifer Allain
Service d'Endocrinologie et Maladies métaboliques
Hôpital d'Instruction des Armées Begin, Saint-Mandé (94)
allain.jennifer@gmail.com
Tél : 01 43 98 50 00

Dr Vincent Melki
Service d'Endocrinologie et Maladies métaboliques et Nutrition
CHU Hôpital de Rangueil, Toulouse (31)
melki.v@chu-toulouse.fr
Tél : 05 61 32 22 42, email

PROMOTEUR

Représentant de MEDTRUM en France :

Mélanie Marmounier
Directeur Commercial et Réglementaire
88 Avenue du Général Leclerc, Boulogne Billancourt
melanie.marmounier@medtrum.com
Tél : 06 20 59 12 24

SOCIETE PRESTATAIRE :

La société prestataire (*Contract Research Organization*, CRO) est chargée de la rédaction du protocole, du cahier d'observation électronique (eCRF), des soumissions réglementaires, de la logistique et du suivi de l'étude, du data management, des analyses statistiques et du rapport clinique.

CRO	**AXONAL-BIOSTATEM S.A.S.**<br>215, avenue Georges Clemenceau<br>92024 Nanterre Cedex, France<br>Tél : + 33 (0)1 56 38 21 50
Chef de Projet	Julien le Dall<br>Tél : +33 (0)1 56 38 36 52<br>Julien.ledall@axonal.com
Directeur de Projet	Alexandra Fursy<br>afursy@axonal.com
Assurance Qualité	Emilie Julien<br>ejulien@axonal.com
Biostatisticienne	Muriel Tounsi<br>muriel.tounsi@biostatem.com
Médecin Projet	Dr Jean-Pierre Meunier<br>jpmeunier@axonal.com

PAGE DE SIGNATURE DE L'INVESTIGATEUR

J'ai lu et approuvé le protocole, version 1.3 du 13/05/2020 intitulé, « **Evaluation de l'intérêt de la pompe patch Medtrum A7+ TouchCare® versus la pompe patch Insulet Omnipod®** ».

Je suis conscient de mes responsabilités en tant qu'Investigateur en vertu des réglementations locales en vigueur et du protocole de l'étude.

J'accepte de mener l'étude en fonction de ces responsabilités et de diriger et d'assister de manière appropriée le personnel sous mon contrôle, qui sera impliqué dans l'étude.

Je discuterai du dispositif médical avec des patients pour m'assurer qu'ils sont pleinement informés sur le dispositif investigué et la conduite de l'étude. Je n'utiliserai que le formulaire de consentement éclairé approuvé par le promoteur et assumerai toutes les responsabilités liées à la présentation de l'information.

J'accepte que les informations confidentielles contenues dans ce document ne soient pas utilisées à d'autres fins que l'évaluation de l'étude clinique sans le consentement écrit préalable de MEDTRUM.

J'accepte que le chargé de recherche / l'attaché de recherche clinique (ARC) et / ou d'autres représentants du Promoteur ou ses mandataires délégués aient accès à toute source de données à partir de laquelle des renseignements sur les cas peuvent avoir été générés, ainsi que tout audit ou inspection.

Nom : ________________________________

Coordonnées : ________________________________

Signature : ________________________________

Date : ________________________________

RESUME DU PROTOCOLE

Titre de l'étude	**Evaluation de l'intérêt de la pompe patch Medtrum A7+ TouchCare® versus la pompe patch Insulet Omnipod®**
Promoteur	MEDTRUM France
Investigateur Principal	Pr Alfred PENFORNIS<br>Chef du service d'Endocrinologie, Diabétologie et Maladies métaboliques du Centre Hospitalier Sud-Francilien de Corbeil-Essonnes (91), France
Comité Scientifique	- Pr Alfred PENFORNIS, service d'Endocrinologie, Diabétologie et Maladies métaboliques du Centre Hospitalier Sud-Francilien de Corbeil-Essonnes (91)<br>- Dr Vincent MELKI, service de Diabétologie, Maladies métaboliques et Nutrition, CHU Hôpital de Rangueil, Toulouse (31)<br>- Dr Jennifer ALLAIN, service d'Endocrinologie et Maladies métaboliques, Hôpital d'Instruction des Armées Begin, Saint-Mandé (94)
Dispositif médical à l'étude	La pompe à insuline du Système de gestion de l'insuline A7+ TouchCare® (MEDTRUM).<br>Marquage CE n° HD 601 357 110001 du 19/02/2019 (TUV Rheinland).<br><br>Alternative au traitement par injections pluri-quotidiennes d'insuline (schéma basal-bolus) pour les patients atteints de diabète de type 1 ou 2 insulino-requérants.
Comparateur	Omnipod® (INSULET) qui est remboursé en France et inscrit sur la LPPR sous nom de marque depuis le 23/02/2016.
Type d'étude	Essai Clinique randomisé en ouvert, avec deux groupes parallèles 1:1, multicentrique national, prospectif, avec une méthodologie de non-infériorité versus le comparateur remboursé et déjà utilisé par le patient pour l'administration de son insuline.
Rationnel de l'étude	*Le traitement des patients atteints de diabète de type 1 et des de type 2 insulino-requérant repose sur une insulinothérapie qui imite la sécrétion physiologique du pancréas par un schéma basal/bolus, obtenu soit par multi-injections soit par pompe. L'objectif de ce schéma basal/bolus est d'approcher la normoglycémie afin de prévenir :*<br>*- à long terme, les complications chroniques du diabète ;*<br>*- À court terme, les complications aiguës du diabète qui sont des urgences métaboliques (y compris le coma) : liées soit à l'hyperglycémie associée à l'acidocétose soit à l'hypoglycémie.*<br>*Le diabète est une maladie grave en raison de ses complications. Cependant, ces complications peuvent être évitées et/ou atténuées grâce à un contrôle métabolique soutenu de la glycémie. Ce contrôle vise à atteindre un taux d'A1C inférieur à 7 ou 7,5% (le pourcentage étant différent selon la recommandation à laquelle on se réfère et le type de patients) sans augmenter les épisodes d'hypoglycémie. Une prise de position de la Société Francophone du Diabète publiée en 2009 précise que depuis des années, les pompes à insuline externes ont prouvé leur efficacité comme traitement intensif du diabète en améliorant le contrôle glycémique et en réduisant les hypoglycémies.*<br><br>*Globalement, les indications d'un traitement par pompe peuvent être résumées comme suit :*<br>*- nécessité d'un programme intensif (au moins 3 injections par jour, 3 auto-surveillances de la glycémie par jour)*<br>*- mauvais contrôle glycémique malgré un traitement intensif (A1C > 7,5 %, 2 épisodes d'hypoglycémie grave ou coma inexpliqué dans l'année et/ou 4 hypoglycémies modérées par semaine)*<br>*- variabilité des besoins en insuline.*<br>*Les contre-indications absolues sont rares et comprennent les troubles psychiatriques graves, la rétinopathie ischémique ou proliférative à progression rapide (avant le traitement au laser) et l'exposition à un champ magnétique élevé.*

	Au cours des décennies actuelles, plusieurs innovations ont vu le jour dans la gestion du diabète. Les pompes sont devenues plus petites, moins invasives et plus faciles à utiliser. Elles offrent également la possibilité d'être dotées de capteurs et d'algorithmes intégrés afin de faire partie d'une boucle qui devrait conduire, à plus long terme, à un pancréas artificiel.<br>*De plus, les modèle les plus récents de pompes dîtes « pompe patch-p » permettent de détecter une occlusion précoce se traduisant par l'absence d'injection d'insuline. Toutes les pompes sont construites avec une alarme d'occlusions, mais elle se déclenche souvent trop tard sur les pompes conventionnelles. Les cathéters longs des pompes conventionnelles ont une certaine élasticité, ce qui signifie qu'ils peuvent se dilater, stocker l'insuline et retarder ainsi le moment d'"hyperpression", qui ne se déclenche que lorsque 5 à 7 unités d'insuline n'ont pas été administrées. Ce retard peut être dangereux, surtout pour les enfants. Dans la nouvelle conception de pompe patch, cette alarme est plus immédiate en cas d'occlusion due à l'absence de cathéter.*<br><br>*Aujourd'hui, en France, il existe deux types de pompes à insuline pour perfusion sous-cutanée continue externe (CSII) :*<br>*1. Les pompes à insuline dites "durables" (également appelées "conventionnelles") qui délivrent de l'insuline en continu à l'aide d'un tube et d'un cathéter externe. Ces modèles figurent sur la LPPR (Liste des Produits et Prestations Remboursables) sur une ligne générique. La LPPR prévoit le remboursement des pompes à insuline et des consommables au moyen de forfaits complets. Les conditions d'enregistrement des pompes à insuline portables et des services associés sont fixées par décret.*<br>*2. Les pompes à insuline externes, appelées "pompes patch", qui ne peuvent être réutilisées et qui sont conçues sans tubulure externe. Ces pompes patch délivrent également de l'insuline en continu, mais le système d'administration d'insuline lui-même n'est pas durable et est géré par une télécommande -un PDM- (Personal Diabetes Manager). Ce type d'appareil ne comprend pas de tubulure externe et ne nécessite pas l'installation d'un cathéter. Les réservoirs d'administration d'insuline adhèrent à la peau à l'aide d'un patch adhésif qui dure 3 jours. Ils sont changés régulièrement mais ne nécessitent pas d'entretien à long terme. L'absence de tubulure réduit le nombre d'incidents de cathéter et de tubulure (tolérance cutanée, obstruction). De plus, ils permettent une détection précoce de l'occlusion et ces systèmes sont plus légers, ce qui permet moins d'encombrement et plus de confort journalier. Ces modèles sont listés sur le LPPR en nom propre (marque).*<br>*La LPPR prévoit le remboursement des pompes à insuline et de services associés à la mise en place, suivi et formation. Les conditions d'enregistrement des pompes à insuline et des services associés sont également fixées par décret.*<br>*Les données épidémiologiques disponibles ne permettent pas de déterminer la population cible de manière précise. Selon les bases de données de la CNAMTS, le nombre moyen de patients utilisant une pompe à insuline externe a été estimé à 41.600 en 2013, avec une augmentation de 18% par rapport à l'année précédente.*<br>*L'administration d'insuline par pompe à un taux de pénétration différent selon le type de diabète et le groupe d'âge (taux de pénétration estimé chez les adultes : 16 %, chez les enfants : 50 %). Cependant, si la pénétration augmente plus rapidement avec les nouveaux appareils, il y a toujours un décrochage chaque année. En effet, si l'on considère les données du SNITEM (Syndicat National de l'Industrie des Technologies Médicales) combinées aux sources ci-dessus, on peut estimer la population de patients traités par pompe à insuline externe à environ 50.000 patients en 2019.*
Objectifs	• Objectif Principal :<br>**Le critère principal d'évaluation est l'estimation de l'HbA1c basée sur la moyenne des mesures du glucose en continu obtenues au cours des 10 dernières semaines pour chaque utilisation de pompe.**<br><br>La moyenne du taux de glucose du patient (relevée sur 10 semaines dans chaque bras) sera calculée à partir des mesures enregistrées automatiquement par un capteur de Mesure Continue du Glucose que le patient utilise déjà : le FreeStyle Libre (Abbott).<br><br>• Objectifs secondaires :<br>o Valeur d'HbA1c mesurée au laboratoire d'analyses médicales et biologiques en début et fin d'étude

	○ Mesures de glucose (minimum, maximum, moyenne/médiane, écart-type, valeur hors plage, temps dans la plage [Time in Range], variabilité)<br>○ Événements glycémiques (hypo et hyperglycémies – selon définition de l'ADA, coma, autres complications)<br>○ Tolérance cutanée et tolérance générale<br>○ Incidents techniques avec le dispositif<br>○ Satisfaction globale des patients, sur la population totale de l'étude, dans chaque bras et la comparaison entre les deux bras<br>○ Conformité du traitement à l'insuline
Population étudiée	**Patients diabétiques de type 1 ou 2 déjà équipés d'une pompe patch à insuline Omnipod® (Insulet) et d'un capteur de glycémie FreeStyleLibre® (Abbott).**
Nombre de patients	75 patients diabétiques (type 1 & 2) recrutés en 3 mois.
Durée du suivi par patient	3 mois à partir de la randomisation. Visites de suivi à 4 semaines puis à 12 semaines.<br>Les patients randomisés dans le groupe contrôle (Omnipod) pourront utiliser s'ils le souhaitent une pompe A7+Touchcare® pendant 1 mois complet après la visite à 3 mois, afin de faciliter le recrutement par les centres et pour permettre une mesure de satisfaction pour les deux pompes.
Nombre de centres	8 centres en France (CHU et CHG).
Critères d'inclusion et d'exclusion	<u>Critères d'inclusion</u><br>• Patient atteint de diabète de type 1 ou 2, âgé de 18 ans et plus<br>• Patient déjà équipé d'une pompe patch à insuline Omnipod® (Insulet) et d'un capteur de glycémie FreeStyleLibre ® (Abbott).<br>• A1C >= 6,5% to <= 9.5%<br>• Traité par tout type d'insuline rapide sauf l'insuline FIASP (qui peut être remplacée au besoin) avec 60 UI maximum par jour (utilisation non autorisée de suppléments d'insuline par injecteur de stylo).<br>• Patient capable de recevoir et de comprendre l'information sur l'étude, de donner son consentement éclairé par écrit et de participer facilement à l'étude.<br><br><u>Critères d'exclusion</u><br>• Patient participant déjà à une autre étude<br>• Patient sous la protection de la justice ou sous tutelle ou curatelle<br>• Patient diabétique de type 2 nécessitant une dose quotidienne d'insuline supérieure à 60 UI par jour<br>• Patient ne pouvant pas continuer l'utilisation d'une pompe à insuline pour des raisons telles que : troubles psychiatriques graves, progression rapide d'une rétinopathie ischémique ou proliférative avant le traitement au laser, exposition à des champs magnétiques<br>• Patient allergique au nickel ou à l'adhésif<br>• Patient non affilié à un régime de sécurité social<br>• Femme enceinte ou femme allaitante<br>• Ou tout autre critère apprécié par l'investigateur.

Critère principal de jugement	**Le critère principal d'évaluation est l'estimation de l'HbA1c à partir de la moyenne des mesures du glucose en continu obtenues au cours des 10 dernières semaines de suivi.**<br><br>Les mesures du glucose seront issues des mesures continues obtenu à l'aide du capteur FreeStyle Libre, et extraites à partir de l'application fournie par le fabricant (déjà utilisée en routine) et reportées par l'investigateur dans l'eCRF.
Critères de jugement secondaires	o Mesures de l'HbA1c à J0 et à 3M (valeurs du laboratoire d'analyse)<br>o Mesures de glucose (minimum, maximum, moyenne/médiane, écart-type, valeurs hors cibles, time in range, variabilité)<br>o Evénements glycémiques (hypo et hyper glycémies -ADA définition, coma, autre complication)<br>o Tolérance de la peau et tolérance globale<br>o Incidents techniques avec le dispositif<br>o Satisfaction globale des patients, sur la population totale de l'étude, dans chaque bras, et comparaison entre les bras<br>o Compliance du traitement à l'insuline
Méthode de Randomisation	Randomisation à l'aide de l'e-CRF.<br>Elle déterminera le type de pompe à utiliser dans l'étude (Omnipod® ou A7+Touchcare®).
Description des dispositifs	La pompe patch Medtrum A7+ TouchCare® est indiquée pour l'administration sous-cutanée continue d'insuline, à des taux fixes et variables, pour la prise en charge du diabète chez les patients insulinodépendants. La pompe est un des éléments d'un système complet : le système de gestion de l'insuline Medtrum A7+ TouchCare®, indiqué pour les patients diabétiques (2 ans et plus). Le système complet comprend (en plus de la pompe) un capteur qui permet la surveillance continue du glucose (CGM) en mesurant le taux de glucose dans le liquide interstitiel. Cette combinaison comprend également des algorithmes qui permettent la mise ne place d'alertes (détection d'hypoglycémie et hyperglycémie) et la suspension automatique de l'administration d'insuline afin de prévenir les épisodes d 'hypoglycémie.<br><br>La pompe A7+ TouchCare® est un dispositif médical de classe IIB.<br><br>Le dispositif médical est composé :<br>- D'une base de pompe contenant les éléments électroniques et d'un système de fixation au réservoir à insuline. Cette base permet le stockage en mémoire des programmes et des doses injectées.<br>- Une « télécommande individuelle » de la pompe (ou *Personal Diabetes Manager* -PDM-) comportant un écran couleur tactile. Le PDM permet de contrôler la pompe et d'administrer l'insuline en continu (transmission sans fil de type radiofréquence). Il permet d'enregistrer les données des 90 derniers jours.<br>- La partie amovible de la pompe est constituée de réservoirs à insuline (consommable adhésif pouvant être porté jusqu'à 3 jours) pouvant contenir 200 unités d'insuline.<br><br>Cette pompe comporte un calculateur de bolus, une technologie utilisée dans les pompes depuis plusieurs années. Il s'agit d'un système déterministe.<br><br>**Les algorithmes de prédiction d'hypoglycémie ou d'hyperglycémie intégrés à la pompe seront désactivés pendant l'étude.**<br><br>Les données de la pompe peuvent être partagées avec les professionnels de santé et les soignants par le biais d'une application mobile (Medtrum EasyTouch®) et d'un portail internet (EasyView®).
Conduite de l'étude et procédures	<u>Procédure de recrutement des sites</u><br>• Faisabilité de l'étude par le centre, validation du potentiel de recrutement de patients en 3 mois, expérience préalable de participation à un essai clinique.

	• Formation sur site par un représentant Medtrum qualifié, sur l'utilisation des 2 modèles de pompes et du capteur fourni aux patients, à l'équipe d'investigation du centre (médecins, infirmières, ARCs).<br><br><u>Procédure de recrutement des patients</u><br>• Proposition aux patients vus en consultation ou à l'hôpital de participer à l'étude s'ils répondent aux critères d'admissibilité à l'étude, avec une notice d'information et de consentement éclairé (voir V0)<br>• Consultation planifiée à court terme après une période de réflexion (7 jours) pour signer le consentement et randomiser la pompe à utiliser par le patient, et pour former le patient à son utilisation (visite V1).<br>• Dans le bras Omnipod, les patients continueront à utiliser leur traitement habituel : leur capteur FreeStyle Libre et leur pompe Omnipod (pour minimiser le risque d'erreur en utilisant 2 modèles identiques de pompe et de capteur).<br>• Dans le bras Medtrum le groupe actif, tous les consommables seront fournis pour la durée de l'étude dans : C'est-à-dire la pompe Medtrum et ses consommables. Ils seront récupérés en fin d'étude.<br><br><u>Chronologie des visites</u><br>• <u>Visite V2 :</u> après 4 semaines d'utilisation de la pompe pour évaluer les résultats et les conditions d'utilisation ;.<br>• <u>Visite V3</u> : après 12 semaines d'utilisation de la pompe pour évaluer les résultats en fin d'étude ;<br>• Les patients du groupe Omnipod pourront utiliser la pompe Medtrum pendant 1 mois après la visite à 3 mois (V3). Cela permettra de mesurer la satisfaction d'utilisation des deux modèles de pompes.<br><br><u>Mesures glycémiques</u><br>Les mesures de glucose du capteur FreeStyle Libre et les informations liées à l'administration de l'insuline obtenues à partir des deux pompes patch seront intégrées dans la base de données de l'étude par l'équipe investigatrice du centre, à l'aide de logiciels fournis par les fabricants.
Données collectées	*Visite d'inclusion (V1)*<br>o Signature du consentement éclairé<br>o Données sociodémographiques<br>o Âge du diabète, type, début de l'insulinothérapie<br>o HbA1c au moment de la prescription de la pompe actuelle<br>o Modèle actuel de la pompe<br>o Satisfaction du patient<br>o Type d'insuline<br>o Dernière mesure d'HbA1c<br>o Paramètres glycémiques<br>o Nombre d'événements glycémiques majeurs (définition de l'ADA) au cours du dernier mois, au cours des 6 derniers mois<br>o Randomisation du modèle de pompe<br>*Première visite de suivi (V2) : 4 semaines*<br>o Tolérance locale et tout événement indésirable

	○ Enregistrement horodaté des valeurs et des événements glycémiques, des hypo et des hyperglycémies, des doses d'insuline administrées.<br>○ Paramètres glycémiques<br>○ Incidents techniques avec le dispositif (occlusion du cathéter, alarmes, détachement, douleur, etc...)<br>○ Satisfaction du patient<br>*Visite de fin d'étude (V3) : 12 semaines*<br>○ Tolérance locale et tout événement indésirable<br>○ Enregistrement horodaté des valeurs et des événements glycémiques, des hypo et des hyperglycémies, des doses d'insuline administrées.<br>○ Paramètres glycémiques<br>○ Incidents techniques avec le dispositif (occlusion du cathéter, alarmes, détachement, douleur, etc...)<br>○ Satisfaction du patient<br><br>*Une période supplémentaire de 1 mois de test pour le groupe Omnipod = visite V4 au 4ème mois pour l'évaluation de leur satisfaction après utilisation de la pompe Medtrum.*<br>○ Tolérance locale et tout événement indésirable<br>○ Enregistrement horodaté des valeurs et des événements glycémiques, des hypo et des hyperglycémies, des doses d'insuline administrées.<br>○ Paramètres glycémiques<br>○ Incidents techniques avec le dispositif (occlusion du cathéter, alarmes, détachement, douleur, etc...)<br>○ Satisfaction du patient
Gestion des vigilances	Complications attendues de l'utilisation de la pompe dans l'étude.<br>Tous les événements survenus au cours de l'étude : incident lié à un instrument médical, événement indésirable grave ou non grave, événement indésirable grave ou non grave lié à une injection d'insuline.
Evaluation des risques	Chaque patient utilise déjà une pompe à insuline au moment de son inclusion dans l'étude. L'utilisation d'un autre modèle de pompe (marqué CE et déjà utilisé dans d'autres pays) ne présente aucun risque potentiel supplémentaire.
Justification du nombre de sujets nécessaire	L'objectif d'une étude de non-infériorité est de démontrer que la différence moyenne entre les 2 dispositifs demeure faible et cliniquement non significative.<br>La glycémie moyenne obtenue dans la vie réelle avec la pompe Omnipod est de 7,8% selon l'expérience des trois centres du comité scientifique de l'étude, ce qui est conforme aux données de la littérature. Nous pouvons supposer que la glycémie moyenne sous La pompe Medtrum est la même que sous la pompe Omnipod.<br>En fixant la borne Δ de non infériorité à +0.4%, conformément aux recommandations de la FDA (Guidance for Industry Diabetes Mellitus (Developing Drugs and Therapeutic Biologics for Treatment and Prevention), si la borne supérieure de l'IC95% de la différence entre les 2 pompes (Medtrum-Omnipod) dépasse Δ (soit 0.4), la non infériorité ne sera pas démontrée.<br>Dans le cas contraire, la supériorité de la pompe Medtrum sur la pompe Omnipod pourra être testée, avec une borne Δ' fixée à -0.3%.

	Sur la base de ces hypothèses, basé sur le design dit du « Less is better » avec α = 2,5%, β = 20% et un écart-type (ET) fixé à 0,55, le calcul du nombre nécessaire de sujets (analysables) est de 60 patients (30 dans chaque groupe).<br>En supposant que 20 % des patients ne peuvent être analysés (écarts majeurs, données manquantes, perdus de vue), **le nombre de patients à randomiser est de 75**. Le nombre de centres à recruter en 3 mois maximum est de 6.<br><br>**Pour confirmer notre hypothèse sur l'écart-type, une analyse descriptive des données de base d'HbA1c pour l'ensemble de la population sera effectuée en aveugle à la fin de la période d'inclusion**. Si une forte différence par rapport à nos hypothèses est trouvée, la taille de l'échantillon sera calculée de nouveau, afin de conserver une puissance statistique de 80 % pour cette étude.
Analyses statistiques	Le traitement des données et les analyses statistiques sera réalisé par la société prestataire, Axonal-Biostatem.<br>Les analyses statistiques seront décrites dans un plan d'analyse statistique (PAS) validé par le Promoteur et le Comité Scientifique, et ce avant le gel de base.<br>Les analyses statistiques seront réalisées après le gel de base des données avec le logiciel SAS® (SAS Institute, NC, Cary, USA), version 9.4 ou ultérieure.<br>Les analyses des données démographiques et d'efficacité/performance seront effectuées dans la population en ITT. Le critère de jugement principal sera analysé en PP (Per Protocol) puis validé sur la population ITT (intention de Traiter). Les analyses de tolérance seront faites dans la population de tolérance.<br>Pour les variables quantitatives, les statistiques usuelles (n, n manquants, moyenne, écart type (ET), médiane, premier et troisième quartiles (Q1 and Q3), minimum et maximum) seront présentées. Les IC95% pourront être présentés si pertinent (notamment sur le critère de jugement principal).<br>Pour les variables qualitatives, les statistiques usuelles (n, n manquants, fréquence et pourcentage) de chaque modalité seront fournies.<br>Les statistiques descriptives seront fournies au global et par groupe de pompes.<br>L'erreur de type 1, α, est fixée à 5% lorsque les tests utilisés seront bilatéraux (supériorité) et α sera fixé à 2.5% lorsque les tests seront unilatéraux dans le cas de la Non infériorité (IC95%).<br>*Analyse de l'objectif principal*<br>L'analyse de non-infériorité sera produite via une Ancova ou un modèle mixte sur la population en Per-Protocol puis en intention de traiter.<br>Dans le cas où la non-infériorité est démontrée, une analyse de supériorité sera conduite.<br>*Analyse des objectifs secondaires*<br>Les critères secondaires seront décrits puis feront l'objet de modèles mixtes ou Ancova.<br>Une analyse de non-infériorité sur les données d'HbA1c et des analyses de supériorité portant sur les Time in range, nombre d'événements glycémiques, satisfaction patients seront réalisées.
Cadre réglementaire	Numéro ID-RCB, avis CPP, CNIL MR001 et compliance RGPD, information auprès de l'ANSM, approbation des contrats investigateurs par le CNOM, obligations transparence
Logistique	CRO Axonal-Biostatem (Nanterre, France)

Calendrier de l'étude	Soumissions réglementaires (CPP) : octobre 2019<br>Estimation date d'obtention de tous les accords : décembre 2019<br>Mise en place des centres : Janvier à octobre 2020<br>Recrutement des patients : Janvier à novembre 2020<br>Dernière visite du dernier patient : mars 2021<br>Gel de base final : juin 2021<br>Résultats sur critère principal : septembre 2021<br>Rapport Clinique validé : décembre 2021<br>Durée totale de participation de chaque centre = 7 mois

ABREVIATIONS

ANSM	Agence Nationale de Sécurité des Médicaments et des produits de santé
ARC	Attaché de Recherche Clinique
ATC	Classification Anatomique, Thérapeutique et Chimique
BPC	Bonnes Pratiques Cliniques (ISO 14155)
BPF	Bonnes Pratiques de Fabrication
CNIL	Commission Nationale de l'Informatique et des Libertés
CNOM	Conseil National de l'Ordre des Médecins
CPP	Comité de Protection des Personnes
CRO	Société Prestataire (*Contract Research Organization*)
eCRF	Cahier d'observation électronique (*Electronic Case Report Form*)
EI	Evénement Indésirable
EIG	Evénement Indésirable Grave
EIGI	Evénement Indésirable Grave Inattendu
ET	Ecart-type
EVA	Echelle Visuelle Analogique
FAS	Full Analysis Set
HAS	Haute Autorité de Santé
HbA1c	Hémoglobine glyquée
ITT	Intention-de-Traiter
J	Jour
M	Mois
MEdDRA	Dictionnaire Médical Réglementaire
NRS	Score d'échelle numérique (de 0 à 10)
PP	Per Protocole
PT	Terme Préférentiel
Q	Quartile
QoL	Qualité de Vie
RGPD	Règlement Général européen sur la Protection des Données (RGPD)
RIPH	Recherche Impliquant la Personne Humaine
SOC	Classification de Système d'Organes
WHO	World Health Organization

SOMMAIRE

PAGE DE VALIDATION DU PROTOCOLE **2**
PAGE DE SIGNATURE DE L'INVESTIGATEUR **5**
RESUME DU PROTOCOLE **6**
ABREVIATIONS **14**
SOMMAIRE **15**
1 INTRODUCTION **17**
1.1 INTRODUCTION ET RATIONNEL DE L'ETUDE 17
2 METHODOLOGIE **19**
2.1 DESIGN DE L'ETUDE 19
2.2 RATIONNEL DU DESIGN DE L'ETUDE 19
3 DISPOSITIF MEDICAL **20**
3.1 IDENTIFICATION ET DESCRIPTION DU DISPOSITIF MEDICAL DE L'ETUDE 20
3.1.1 Mécanisme d'action d'une pompe à insuline *20*
3.1.2 Dispositif médical à l'étude : Pompe patch à insuline A7+ TouchCare Medtrum *20*
3.1.3 Procédure d'utilisation du dispositif *23*
3.1.4 Événements indésirables possibles liés à l'utilisation du dispositif *23*
3.1.5 Conditionnement et étiquetage *24*
3.1.6 Conservation et stockage *24*
3.1.7 Approvisionnement des dispositifs *24*
3.1.8 Randomisation *24*
3.1.9 Dispensation des dispositifs *24*
3.1.10 Retour des dispositifs *24*
3.2 OBSERVANCE 25
3.3 EFFETS INDESIRABLES GRAVES ATTENDUS 25
3.4 AUTRES DISPOSITIFS UTILISES DANS L'ETUDE 25
3.4.1 Lecteur de glycémie en continu FreeStyle Libre *25*
3.4.2 Patch pompe Insulet Omnipod® (dispositif comparateur) *26*
3.4.3 Centralisation des sources d'information et clés d'identification *26*
4 OBJECTIFS DE L'ÉTUDE **27**
4.1 OBJECTIF PRINCIPAL 27
4.2 OBJECTIFS SECONDAIRES 27
5 SELECTION DES PATIENTS **28**
6 POPULATION DE L'ETUDE **28**
6.1 CRITERES D'INCLUSION 28
6.2 CRITERES DE NON-INCLUSION 29
6.3 TRAITEMENTS PROHIBES 29
6.4 CRITERES DE SORTIE PREMATUREE DU PATIENT DE L'ETUDE 29
6.5 REMPLACEMENTS 29
7 CALENDRIER DES VISITES ET PROCEDURES D'ETUDE **30**
7.1 CALENDRIER DES VISITES 30
7.2 CALENDRIER DES EVALUATIONS 30
7.3 VISITE 1 : VISITE D'INCLUSION AVEC RECUEIL DU CONSENTEMENT ECLAIRE 31
7.4 VISITE 2 (4 SEMAINES) : VISITE DE SUIVI APRES 4 SEMAINES D'UTILISATION DE LA POMPE 31
7.5 VISITE 3 (12 SEMAINES) : VISITE DE FIN D'ETUDE 31
7.6 VISITE 4 (4 SEMAINES APRES FIN D'ETUDE) : APRES 1 MOIS D'UTILISATION DE LA POMPE MEDTRUM (UNIQUEMENT PATIENTS RANDOMISES DANS LE GROUPE OMNIPOD) 32
8 QUESTIONNAIRES PATIENT **32**
9 COLLECTE ET CONTROLE QUALITE DES DONNEES **33**

9.1 SYSTEME D'INFORMATION 33
9.2 SAISIE DES DONNEES 33
9.2.1 Saisie par l'investigateur 33
9.2.2 Saisie par le patient 34
9.3 CONTROLE QUALITE DES DONNEES 34
9.4 GEL FINAL DE LA BASE DE DONNEES 34

10 CRITERES D'EVALUATION ET DE SUIVI 34

10.1 CRITERE PRINCIPAL D'EVALUATION 34
10.2 CRITERES SECONDAIRES D'EVALUATION 35
10.3 AUTRES CRITERES 36

11 ANALYSE STATISTIQUE 36

11.1 JUSTIFICATION DU NOMBRE NECESSAIRE DE PATIENTS 36
11.2 ANALYSE STATISTIQUE 37
11.2.1 Méthodes statistiques générales 37
11.2.2 Populations étudiées 37
11.2.3 Analyse Descriptive 38
11.2.4 Analyse de l'efficacité/performance du dispositif 38
11.2.5 Analyses de la tolérance : 40
11.2.6 Analyse intermédiaire 40
11.2.7 Analyses en sous-groupes, analyses exploratoires 40

12 CONTROLES QUALITE 40

13 EFFETS INDESIRABLES, VIGILANCE 41

13.1 DEFINITIONS 42
13.2 RESPONSABILITES DE L'INVESTIGATEUR 44
13.2.1 Notification des évènements indésirables (EI) 44
13.2.2 Notification des Evénements indésirables graves (EIG) 45
13.2.3 Notification des grossesses 46
13.2.4 Notification des surdosages 47
13.2.5 Notification des Faits nouveaux 47
13.2.6 Notification des incidents de vigilance 47
13.3 RESPONSABILITES DU PROMOTEUR 48
13.4 COMITE DE SURVEILLANCE 48

14 CONSIDERATIONS ETHIQUES ET LEGALES 49

14.1 CADRE REGLEMENTAIRE DE L'ETUDE 49
14.2 SOUMISSION DU PROTOCOLE ET DU CONTRAT D'ETUDE 49
14.2.1 Déclaration aux Autorités Réglementaires Compétentes 49
14.2.2 Déclaration au Comité d'éthique 49
14.2.3 Protection des données à caractère personnel 49
14.2.4 Déclaration aux Ordres Professionnels 50
14.3 INFORMATION ET CONSENTEMENT DU PATIENT 50
14.4 CONFIDENTIALITE 50
14.5 ARRET DE L'ETUDE 51
14.6 ARCHIVAGE 51
14.7 ASSURANCE ET FINANCEMENT 51

15 DOCUMENTATION ET UTILISATION DES RESULTATS DE L'ETUDE 52

16 CALENDRIER DE L'ETUDE 52

17 REFERENCES BIBLIOGRAPHIQUES 53

18 ANNEXES 56

18.1 ASSURANCE DE L'ETUDE 56

Etude « MEDINPS » : Evaluation de l'intérêt de la pompe patch Medtrum A7+ TouchCare® versus la pompe patch Insulet Omnipod®

1 INTRODUCTION

1.1 INTRODUCTION ET RATIONNEL DE L'ETUDE

Le traitement des patients atteints de diabète de type 1 et de certains diabétiques de type 2 repose sur une insulinothérapie qui imite la sécrétion physiologique du pancréas par un schéma basal/bolus, obtenu soit par pluri-injections quotidienne soit par pompe à insuline externe. L'objectif de ce schema basal/bolus est d'approcher la normoglycémie afin de prévenir :

- à long terme, les complications chroniques du diabète ;

- à court terme, les complications aiguës du diabète qui sont des urgences métaboliques (y compris le coma) : liées soit à l'hyperglycémie et à l'acidocétose soit à l'hypoglycémie.

Le diabète est une maladie grave en raison de ses complications. Cependant, ces complications peuvent être évitées et/ou atténuées grâce à un contrôle métabolique soutenu de la glycémie. Ce contrôle vise à atteindre un taux d'A1C inférieur à 7 ou 7,5% (le pourcentage étant différent selon la recommandation à laquelle on se réfère et le type de patients) sans augmenter les épisodes d'hypoglycémie. Une prise de position de la Société Francophone du Diabète publiée en 2009[14] précise que depuis des années, les pompes à insuline externes ont prouvé leur efficacité comme traitement intensif du diabète en améliorant le contrôle glycémique et en réduisant les hypoglycémies.

Globalement, les indications d'un traitement par pompe peuvent être résumées comme suit :

- nécessité d'un programme intensif (au moins 3 injections par jour, 3 auto-surveillances de la glycémie par jour)

- mauvais contrôle glycémique malgré un traitement intensif (A1C > 7,5 %, 2 épisodes d'hypoglycémie grave ou coma inexpliqué dans l'année et/ou 4 hypoglycémies modérées par semaine)

- variabilité des besoins en insuline.

Les contre-indications absolues sont rares et comprennent les troubles psychiatriques graves, la rétinopathie ischémique ou proliférative à progression rapide (avant le traitement au laser) et l'exposition à un champ magnétique élevé.

Au cours des décennies actuelles, plusieurs innovations ont vu le jour dans la gestion du diabète. Les pompes sont devenues plus petites, moins invasives et plus faciles à utiliser. Elles offrent également la possibilité d'être dotées de capteurs et d'algorithmes intégrés afin de faire partie d'une boucle qui devrait conduire, à plus long terme, à un pancréas artificiel.

De plus, les modèle les plus récents de pompes dîtes « pompe patch » permettent de détecter une occlusion précoce se traduisant par l'absence d'injection d'insuline. Toutes les pompes sont construites avec une alarme d'occlusions, mais elle se déclenche souvent trop tard sur les pompes conventionnelles. Les cathéters longs des pompes conventionnelles ont une certaine élasticité, ce qui signifie qu'ils peuvent se dilater, stocker l'insuline et retarder

ainsi le moment d'"hyperpression", qui ne se déclenche que lorsque 5 à 7 unités d'insuline n'ont pas été administrées. Ce retard peut être dangereux, surtout pour les enfants. Dans la nouvelle conception de pompe patch, cette alarme est plus immédiate en cas d'occlusion due à l'absence de cathéter[15].

Aujourd'hui, en France, il existe deux types de pompes à insuline pour perfusion sous-cutanée continue externe (CSII)[16] :

1. Les pompes à insuline dites "durables" (également appelées "conventionnelles") qui délivrent de l'insuline en continu à l'aide d'un tube et d'un cathéter externe. Ces modèles figurent sur la LPPR (Liste des Produits et Prestations Remboursables) sur une ligne générique. La LPPR prévoit le remboursement des pompes à insuline et des consommables au moyen de forfaits complets. Les conditions d'enregistrement des pompes à insuline portables et des services associés sont fixées par décret.

2. Les pompes à insuline externes, appelées "pompes patch", qui ne peuvent être réutilisées et qui sont conçues sans tubulure externe. Ces pompes patch délivrent également de l'insuline en continu, mais le système d'administration d'insuline lui-même n'est pas durable et est géré par une télécommande -un PDM- (Personal Diabetes Manager). Ce type d'appareil ne comprend pas de tubulure externe et ne nécessite pas l'installation d'un cathéter. Les réservoirs d'administration d'insuline adhèrent à la peau à l'aide d'un patch adhésif qui dure 3 jours. Ils sont changés régulièrement mais ne nécessitent pas d'entretien à long terme. L'absence de tubulure réduit le nombre d'incidents de cathéter et de tubulure (tolérance cutanée, obstruction). De plus, ils permettent une détection précoce de l'occlusion et ces systèmes sont plus légers, ce qui permet moins d'encombrement et plus de confort journalier. Ces modèles sont listés sur le LPPR en nom propre (marque). La LPPR prévoit le remboursement des pompes à insuline de services associés à la mise en place, suivi et formation. Les conditions d'enregistrement des pompes à insuline et des services associés sont également fixées par décret.

Les données épidémiologiques disponibles ne permettent pas de déterminer la population cible de manière précise. Selon les bases de données de la CNAMTS, le nombre moyen de patients utilisant une pompe à insuline externe a été estimé à 41.600 en 2013, avec une augmentation de 18% par rapport à l'année précédente.

L'administration d'insuline par pompe à un taux de pénétration différent selon le type de diabète et le groupe d'âge (taux de pénétration estimé chez les adultes : 16 %, chez les enfants : 50 %). Cependant, si la pénétration augmente plus rapidement avec les nouveaux appareils, il y a toujours un décrochage chaque année. En effet, si l'on considère les données du SNITEM (Syndicat National de l'Industrie des Technologies Médicales) combinées aux sources ci-dessus, on peut estimer la population de patients traités par pompe à insuline externe supérieure à 50.000 patients en 2019[16]. Des données qui restent à confirmer et affiner à la lumière du Rapport Charges et Produits 2020.

2 METHODOLOGIE

2.1 DESIGN DE L'ETUDE

Il s'agit d'une étude interventionnelle longitudinale, **comparative randomisée**, portant sur des patients diabétiques de type 1 ou 2, nécessitant un traitement par insuline délivré par une pompe patch. L'étude est multicentrique en France, prospective, randomisée en deux groupes parallèles 1:1, en ouvert, avec une méthodologie de non-inferiorité versus un dispositif comparateur (pompe Omnipod® commercialisée par la société Insulet et qui est déjà remboursée en France depuis 23/02/2016).

75 patients diabétiques de type 1 ou 2 seront inclus dans l'étude par 8 centres hospitaliers CHU ou CHG spécialisés de diabétologie.

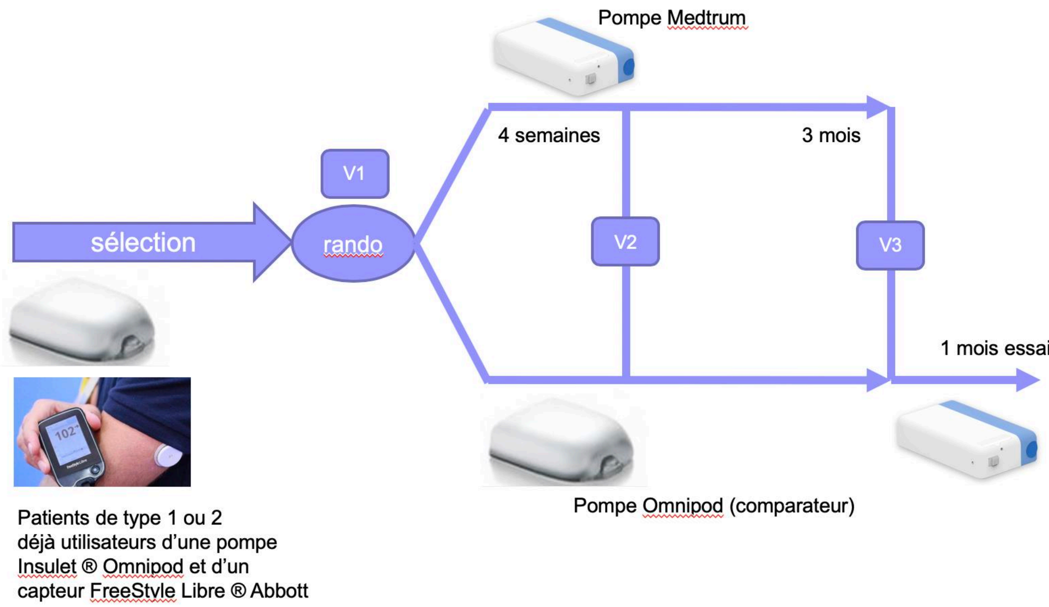

Afin de faciliter le recrutement de patients déjà utilisateurs d'une pompe Omnipod® qui seraient randomisés dans le groupe Omnipod (donc sans changement de leur pompe) il est proposé que ces patients puissent utiliser une pompe Medtrum en fin d'étude pendant 1 mois. Ainsi tous les patients de l'étude auront la possibilité d'utiliser la nouvelle pompe A7+ TouchCare®.

La durée de l'étude sera : 1 mois de mise en place, 3 mois de recrutement des patients, 12 semaines de suivi par patient, 1 mois de plus pour les patients du groupe Omnipod pour tester la pompe Medtrum, 3 mois de gestion des données soit environ 11 mois d'étude.

2.2 RATIONNEL DU DESIGN DE L'ETUDE

Les objectifs de cette étude sont de générer des données spécifiques sur l'usage de la pompe à insuline modèle A7+ TouchCare® de la société MEDTRUM (dispositif médical marqué CE), de collecter des données de sécurité, de tolérance, de performance.

3 DISPOSITIF MEDICAL

3.1 IDENTIFICATION ET DESCRIPTION DU DISPOSITIF MEDICAL DE L'ETUDE

3.1.1 Mécanisme d'action d'une pompe à insuline

La pompe à insuline externe est un appareil discret qui délivre continuellement de petites quantités d'insuline rapide, grâce à une programmation de son utilisateur et un dispositif de perfusion externe et déconnectable (cathéter et tubulure) à changer régulièrement.

Grâce à une programmation de son utilisateur, la pompe à insuline reproduit ce que l'organisme fait naturellement :

- Délivrer en continu de petites doses d'insuline rapide, à intervalles régulier, (débit basal) tout au long de la journée.
- Délivrer une dose supplémentaire d'insuline (bolus) adaptée au moment des repas pour couvrir les glucides absorbés à cette occasion, lors d'une collation ou pour corriger une hyperglycémie.

Ainsi, la pompe à insuline est une alternative au traitement par multi-injections d'insuline réalisées avec les stylos et favorise un meilleur équilibre glycémique.

Les pompes dites « patch » sont des pompes sans cathéter ni tubulure externe, qui sont fixées directement sur la peau à l'aide d'un adhésif.

3.1.2 Dispositif médical à l'étude : Pompe patch à insuline A7+ TouchCare Medtrum

La pompe patch à insuline A7+ TouchCare® (dispositif médical du fabricant MEDTRUM) qui sera utilisée dans l'étude est la version ayant obtenu le marquage CE n° HD 601 357 110001 en date du 19/02/2019 (Organisme Notifié TUV Rheinland).

Figure 1. Aspect de la pompe A7+ TouchCare®

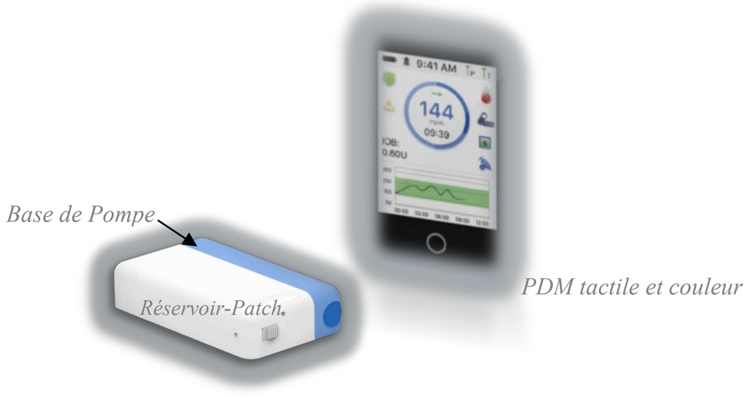

La pompe A7+ TouchCare® est un dispositif médical de classe IIB.

Le dispositif médical est composé :

D'une unité d'administration d'insuline qui requiert 2 éléments :

- La base de pompe, qui est un élément durable du système contenant les éléments électroniques. Véritable mémoire de l'unité d'administration de l'insuline elle permet la mémorisation des programmes et des doses injectées. Elle se fixe à un consommable, le Réservoir Patch
- Le Réservoir Patch est la partie amovible de la pompe qui contient le réservoir à insuline. Il est doté d'une aiguille (canule) destinée à administrer l'insuline. C'est un consommable adhésif pouvant être porté jusqu'à 3 jours et contenir jusqu'à 200 unités d'insuline.

- Une Télécommande individuelle de la pompe (ou *Personal Diabetes Manager* -PDM) comportant un écran couleur tactile. Le PDM permet de contrôler la pompe et d'administrer l'insuline en continu (transmission sans fil de type radiofréquence). Il permet d'enregistrer les données sur les 90 jours précédents.

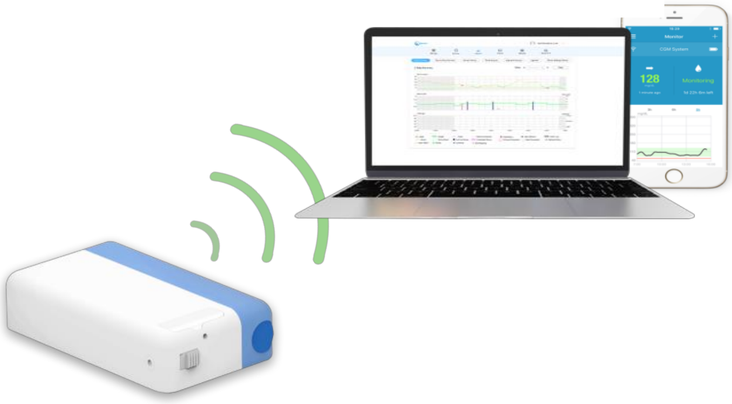

Cette pompe comporte un calculateur de bolus. L'algorithme est une technologie utilisée dans les pompes depuis plusieurs années. Il s'agit d'un système déterministe.

Les algorithmes de prédiction d'hypoglycémie ou d'hyperglycémie intégrés à la pompe seront désactivés pendant l'étude.

Les données de la pompe peuvent être partagées avec les professionnels de santé et les soignants par le biais d'une application sur ordinateur (Medtrum EasyTouch®) et d'un portail internet (EasyView®).

Description détaillée de la pompe A7+ TouchCare®

La pompe patch Medtrum A7+ TouchCare® est indiquée pour l'administration continue d'insuline par voie sous-cutanée, à des taux fixes et variables, pour la prise en charge du diabète chez les patients insulinodépendants. La pompe est un des éléments d'un système complet appelé **Medtrum A7+ TouchCare® Insulin Management System**, indiqué pour les patients diabétiques (2 ans et plus). Le système complet combine un dispositif de surveillance

continue du glucose (CGM) indiqué pour la surveillance continue du taux de glucose dans le liquide interstitiel et la détection d'éventuels épisodes d’hypoglycémie ou d’hyperglycémie ainsi qu’un système d’arrêt automatique de l’insuline destiné à la prévention de l’hypoglycémie.

La pompe patch Medtrum A7+ TouchCare® en tant que pompe autonome est conçue pour l'administration sous-cutanée continue d'insuline. Cette pompe patch est composée de deux éléments principaux et de trois dispositifs médicaux :

Unité d’administration d’insuline

Base de pompe

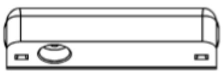

Réservoir Patch

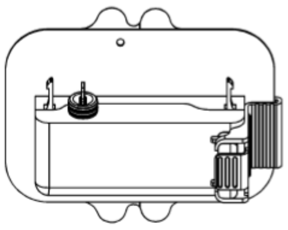

PDM

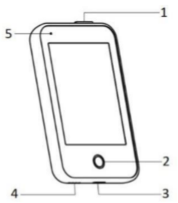

1. Bouton d'alimentation
2. Touche d'accueil
3. Port de chargement
4. Orifice de sortie de son
5. Voyant

✓ Gestionnaire Personnel de Diabète (FM-018)
(Personal Diabetes Manager, PDM)

<u>**1/ Unité d’administration de l’insuline**</u> résulte de la combinaison de 2 dispositifs :

- Une partie durable <u>dite</u> **base de pompe** qui contient l'électronique et mémorise la programmation de l’unité d’administration d’insuline. Elle et doit être fixée au consommable, sur le Réservoir-Patch pour permettre l'administration d'insuline.
- Les **Réservoirs-Patch** sont donc les consommables. Ces derniers sont jetables et contiennent jusqu'à 200 unités d'insuline rapide. Le réservoir du dispositif A7+ se changera tous les 3 jours.

C’est donc la combinaison de ces deux éléments qui permet l’administration continue de l’insuline grâce un système sans tubulure ni cathéters externes.
Ensuite, pour gérer la pompe, le patient devra se munir :

2/La télécommande ou <u>***Personal Diabetes Manager* (PDM)**</u> dispose d’un écran tactile couleur. Le PDM permet la programmation et le contrôle la pompe (et le système de surveillance continue de la glycémie le cas échéant) via une communication radiofréquence (RF) sans fil. Le PDM stocke egalement les données de la pompe (et du capteur) sur une

période pouvant atteindre 90 jours. Le PDM se recharge sur secteur et ne nécessite pas de piles.

La pompe Medtrum A7+ TouchCare® (par rapport à la pompe Insulet Omnipod®) est un élément autonome qui fait partie d'un système de gestion de l'insuline complet. Système qui fournit également la mesure du glucose dans le liquide interstitiel et un la possibilité de mettre en place des alarmes de prévention de l'hyper et l'hypoglycémie ainsi qu'une suspension prédictive de l'administration d'insuline pour éviter les hypoglycémies..
Elle offre également aux patients de la souplesse et notamment la possibilité de personnaliser le débit d'insuline, voire de le réduire à 0 unité/heure, ce qui permet son utilisation notamment en pédiatrie. De plus, la pompe Medtrum A7+ TouchCare® est livrée avec un système de gestion basé sur le cloud qui peut être utilisé à partir d'une application et d'un ordinateur portable fournissant des informations en temps réel aux soignants et permettant un suivi du patient à distance.

Table 1. Caractéristiques administratives du Dispositif médical à l'étude

Nom du produit à l'étude	Pompe à insuline A7+ TouchCare®
Indication	Diabète insulino requérant
Présentation / Composition	Base de pompe permanente associée à son consommable, le Réservoir Patch d'insuline (jetable)<br>Télécommande individuelle (PDM)
Classe	Classe IIB
Titulaire du Dispositif médical	MEDTRUM
Marquage CE	19/02/2019

3.1.3 Procédure d'utilisation du dispositif

Une formation initiale de chaque centre à l'utilisation du dispositif Medtrum sera réalisée par un représentant habilité du fabricant.

Medtrum met à disposition des médecins un système d'exploitation des données via un portail en ligne permettant de visualiser les données recueillies par la pompe A7+ TouchCare®. Les centres investigateurs pourront extraire les données pour les besoins de l'étude (données reportées sur l'eCRF), comme ils le font déjà pour les données d'une pompe Omnipod® et celles d'un capteur Abbott FreeStyle Libre.

3.1.4 Événements indésirables possibles liés à l'utilisation du dispositif

Les investigateurs devront déclarer tout événement se produisant pendant l'étude (cf. chapitre 13).

De problèmes de tolérance sont déjà rapportés pour les patchs des capteurs et pompes à insuline déjà commercialisés sur le marché. Il s'agit essentiellement d'événements à type d'allergie locale de la peau à l'emplacement de l'adhésif de fixation de la pompe.

D'autres effets secondaires dont la gravité et l'aspect ne sont pas connus à ce jour pourraient apparaître et seront donc renseignés pendant l'étude.

3.1.5 Conditionnement et étiquetage

Les étiquettes seront préparées conformément aux bonnes pratiques de fabrication (BPF) et aux exigences réglementaires des essais cliniques, afin d'assurer une traçabilité complète de l'utilisation des dispositifs du début à la fin de l'étude. Les dispositifs seront numérotés individuellement de façon unique.

3.1.6 Conservation et stockage

Les dispositifs de l'étude devront être conservés à température ambiante, et dans un endroit sécurisé dans des conditions de stockage appropriées.

3.1.7 Approvisionnement des dispositifs

Les dispositifs de l'étude seront fournis par le promoteur et adressés à chaque centre (pharmacien hospitalier) qui accusera de leur bonne réception.

3.1.8 Randomisation

La randomisation sera effectuée à l'aide de l'eCRF mis à disposition des investigateurs pour l'étude. Elle déterminera le bras dans lequel sera affecté le patient : soit groupe « Omnipod » (le patient poursuivra l'utilisation de sa pompe actuelle Omnipod) soit dans le groupe « Medtrum » (le patient devra changer de pompe au profit d'un modèle Medtrum).

3.1.9 Dispensation des dispositifs

Les dispositifs à l'étude fournis seront utilisés uniquement comme indiqué dans le protocole de l'étude. Le personnel du site de l'étude sera responsable de tous les dispositifs délivrés au patient.

La date de dispensation, le numéro d'identification du patient, le numéro de lot du dispositif médical devront être enregistrés dans les sections appropriées du dossier-investigateur. L'étiquette de traçabilité sera conservée dans le dossier-investigateur ou le dossier médical du patient.

L'équipe médicale de l'investigateur assurera une formation initiale à l'utilisation de la pompe pour chaque patient et remettra la documentation associée.

Les patients inclus dans l'étude seront déjà utilisateurs des autres dispositifs utilisés dans le cadre de l'étude (pompe Omnipod® et lecteur de glycémie FreeStyle libre), et les utiliseront dans le cadre de l'étude. Aucune intervention sur l'insuline et son administration n'est prévue dans le cadre du protocole.

3.1.10 Retour des dispositifs

Les dispositifs de l'étude non utilisés ne devront pas être jetés ou utilisés à d'autres fins que la présente étude. Ils devront être conservés dans leur emballage d'origine.

L'attaché de recherche clinique (ARC) en charge du monitoring recueillera en fin d'étude les formulaires de distribution des dispositifs médicaux à l'étude et vérifiera tous les retours avant de prendre des dispositions nécessaires pour rapatriement direct au Promoteur ou son prestataire.

3.2 OBSERVANCE

La délivrance du traitement par insuline sera réalisée par la pompe, et les doses déterminées par le patient et injectées par la pompe sont enregistrées par le dispositif. Ces données sont ensuite visualisables et téléchargeables par le centre à l'aide de l'application en ligne mise à disposition par le fabricant.
Une mesure de l'observance des mesures continue du glucose réalisées par le patient sera recueillie à l'aide des données enregistrées dans le dispositif FreeStyle Libre.

3.3 EFFETS INDESIRABLES GRAVES ATTENDUS

Comme pour tout dispositif médical de classe IIB on ne peut exclure des risques liés à l'utilisation.

Chaque patient sera déjà utilisateur d'une pompe à insuline Omnipod® au moment de son inclusion dans l'étude. L'utilisation d'un autre modèle de pompe (marqué CE et déjà utilisé dans d'autres pays) ne présente aucun risque potentiel supplémentaire.

Tout événement indésirable grave, lié ou non au dispositif à l'étude ou à son utilisation, doivent être impérativement déclarées conformément aux instructions figurant au chapitre 13.

3.4 AUTRES DISPOSITIFS UTILISES DANS L'ETUDE

Dans le cadre de cette étude, il sera remis au patient en plus de la pompe A7+ TouchCare® Medtrum les éléments suivants :

3.4.1 Lecteur de glycémie en continu FreeStyle Libre

Le lecteur FreeStyle Libre est un système de Mesure Continue du Glucose (MCG) destinée aux patients diabétiques (dispositif médical de classe IIb). Il affiche les données de taux de glucose recueillies par le capteur associé qui mesure les taux de glucose dans le liquide interstitiel. Contrairement aux CGM déjà existant sur le marché, le FreeStyle LIbre utilise la technologie NFC et requiert que le capteur soit scanné régulièrement pour un relevé continue des taux de glucose.

Il enregistre et mémorise au maximum 90 jours de données de taux de glucose. Pour obtenir une visualisation complète des taux de glucose sur les 3 derniers mois, le capteur doit être remplacé tous les 14 jours et être scanné par le patient au moins une fois toutes les 8 heures. Le capteur doit être retiré avant de subir une IRM.

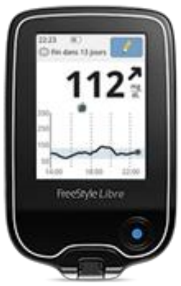

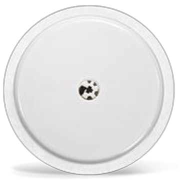

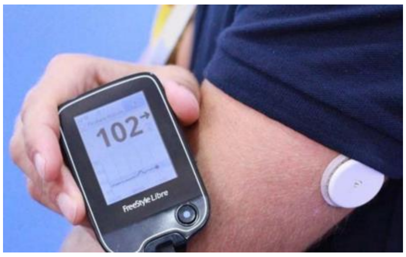

Les fonctions de ce lecteur sont les suivantes :

- Mesure du glucose en continu sur le liquide interstitiel grâce à un capteur posé sur l'épiderme à l'aide d'une base adhésive (durée d'utilisation maximum 14 jours)
- Affichage des données sur un Lecteur (Terminal portable sans fil) de visualisation des données par le patient. L'application FreeStyle LibreLink est également disponible c'est un dispositif médical qui permet de remplacer le lecteur FreeStyle Libre. Il est possible de scanner le capteur FreeStyle Libre en utilisant soit l'application LibreLink sur Android ou iOS, soit le lecteur FreeStyle Libre, soit les deux.
- Les données du système peuvent être téléchargées sur un ordinateur puis transmises dans le cloud du fabricant pour que le médecin puisse consulter les informations

Le fabricant Abbott met à disposition des médecins une application en ligne permettant de visualiser les données recueillies par le lecteur FreeStyle Libre. Les centres investigateurs déjà équipés de ce logiciel pourront extraire les données pour les besoins de l'étude (données à reporter sur l'eCRF).

3.4.2 Patch pompe Insulet Omnipod® (dispositif comparateur)

Ce dispositif médical est composé d'une pompe, de petite taille, qui se colle directement sur la peau et l'insuline est administrée dans le tissu sous-cutané au travers d'une canule qui pénètre la peau lors de l'amorçage de la pompe. L'insuline est injectée directement dans le réservoir intégré à la pompe (pas de réservoir séparé).
La pompe doit être retirée lorsqu'elle est vide, au maximum tous les 3 jours, et éliminée, une nouvelle étant mise en place pour la suite du traitement. C'est donc une pompe à usage unique.

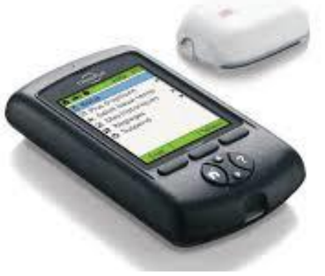

La pompe dispose d'une commande individuelle (ou *Personal Diabetes Manager :* PDM) comportant un écran non tactile. Le PDM permet de contrôler la pompe et d'administrer l'insuline en continu (transmission sans fil de type radiofréquence). Il permet d'enregistrer les données sur les 90 jours précédents.

3.4.3 Centralisation des sources d'information et clés d'identification

La base de données cliniques de l'étude sera gérée exclusivement par le prestataire en charge de la logistique de l'étude (Axonal-Biostatem). Cette base ne comportera aucune donnée directement nominative.
Le prestataire met à disposition des centres un cahier d'observation électronique (e-CRF) accessible par internet. Chaque utilisateur est identifié par un login/password personnel et unique.

Les identifiants de chaque patient selon les différentes sources de données sont les suivants :

- **ID-pompe :** numéro de kit pompe remis au patient. Cet identifiant sera saisi dans la base clinique sur le format |__|__|__|.
- **ID-eCRF** : identifiant unique du patient pour l'étude dans la base de données cliniques (eCRF) sur le format n° de centre – n° de patient au format |__|-|__|__|.

Les centres utiliseront également des applications accessibles par internet, mis à disposition des fabricants pour visualiser et exploiter les données enregistrées et transmises par les différents dispositifs utilisés (pompe, lecteur de glycémie).
A titre d'exemple, les données sont restituées au médecin sous forme de graphiques, tableaux et valeurs moyennes calculées. Les médecins pourront ainsi disposer pour chaque patient, des glycémies moyennes sur les périodes définies, l'estimation de l'A1C, le % de time in range, etc.

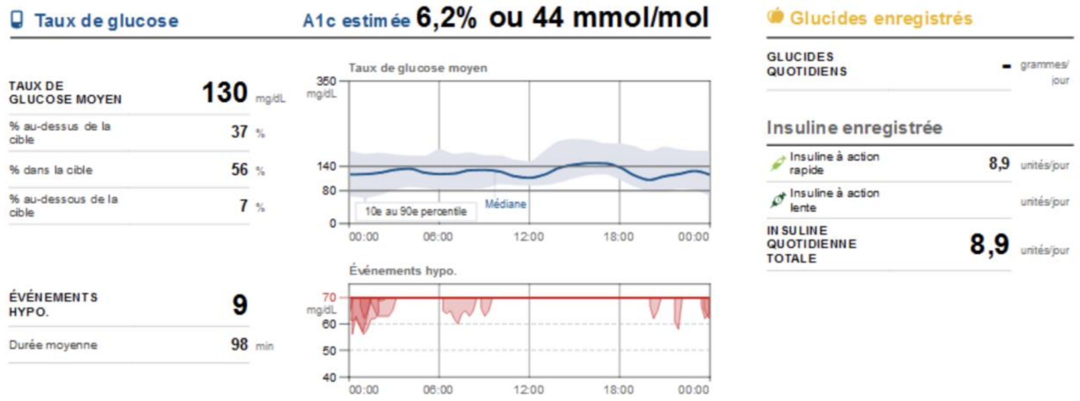

Ces applications sont utilisées en routine dans les centres. Seule l'application fournie par Medtrum pour les données de sa pompe devra faire l'objet d'une formation spécifique des centres.
Les investigateurs devront reporter ces variables calculées dans l'eCRF pour alimenter la base clinique de l'étude.

4 OBJECTIFS DE L'ÉTUDE

4.1 OBJECTIF PRINCIPAL

Le critère principal d'évaluation est l'estimation de l'HbA1c basée sur les mesures moyennes de glycémie au cours des 10 dernières semaines pour chaque utilisation de pompe.

La glycémie moyenne du patient sur les 10 dernières semaines sera calculée dans chaque bras à partir des mesures enregistrées automatiquement par un capteur FreeStyleLibre, que le patient utilise déjà.

4.2 OBJECTIFS SECONDAIRES

Les objectifs secondaires sont de décrire les informations suivantes :

- Valeur d'HbA1c mesurée au laboratoire d'analyses médicales et biologiques en début et fin d'étude
- Mesures du glucose (minimum, maximum, moyenne/médiane, écart-type, valeur hors cible, temps dans la plage [Time in Range], variabilité)
- Événements glycémiques (hypoglycémies et hyperglycémies – selon définition de l'ADA[25], coma, autres complications)
- Tolérance cutanée et tolérance générale
- Incidents techniques avec le dispositif
- Satisfaction globale des patients, sur la population globale, dans chaque bras et comparaison entre les deux bras
- Conformité du traitement à l'insuline

5 SELECTION DES PATIENTS

Les centres investigateurs proposeront la participation à l'étude pour chaque patient potentiellement éligible à l'étude selon les critères d'inclusion et de non-inclusion.

Chaque patient devra être informé par l'investigateur oralement et par écrit au moyen de la notice d'information leur communiquant les objectifs et les modalités de l'étude, du recueil et du traitement informatique des données de santé les concernant, de leur droit de retrait de l'étude sans avoir à se justifier. Le patient aura la possibilité de poser des questions au personnel d'étude du centre investigateur. Si un patient décide de participer, il doit signer en connaissance de cause le formulaire de consentement approuvé par le Comité d'éthique, avant toute procédure liée à l'étude.

Un patient ne sera inclus dans l'étude clinique qu'après avoir donné son consentement éclairé et écrit, et satisfait à tous les critères d'inclusion et à aucun des critères de non-inclusion.

Les patients de cette étude n'étant pas concernés par un traitement médical d'urgence, aucun consentement ne sera collecté dans ce cadre spécifique de l'urgence.

6 POPULATION DE L'ETUDE

6.1 CRITERES D'INCLUSION

Pour être inclus dans l'étude, tous les patients devront satisfaire tous les critères d'inclusion suivants :

1. Patient atteint de diabète de type 1 ou 2, âgé de 18 ans et plus
2. Patient déjà équipé d'une pompe patch à insuline Omnipod® (Insulet) et d'un capteur de glycémie FreeStyleLibre sensor (Abbott).
3. A1C comprise entre ≥6,5% - ≤ 9,5%

4. Traité par tout type d'insuline rapide sauf l'insuline FIASP (qui peut être remplacée au besoin) avec 60 UI maximum par jour (utilisation non autorisée de suppléments d'insuline par injecteur de stylo).
5. Patient capable de recevoir et de comprendre l'information sur l'étude, de donner son consentement éclairé par écrit et de participer facilement à l'étude.

6.2 CRITERES DE NON-INCLUSION

Les patients ne participeront pas à cette étude clinique s'ils répondent **à au moins l'un des critères suivants** :

1. Patient participant déjà à une autre étude
2. Patient sous la protection de la justice ou sous tutelle ou curatelle
3. Patient diabétique de type 2 nécessitant une dose quotidienne d'insuline supérieure à 60 UI par jour
4. Patient ne pouvant pas continuer l'utilisation d'une pompe à insuline pour des raisons telles que : troubles psychiatriques graves, progression rapide d'une rétinopathie ischémique ou proliférative avant le traitement au laser, exposition à des champs magnétiques)
5. Patient allergique au nickel ou à l'adhésif
6. Patient non affilié à un régime de sécurité social
7. Femme enceinte et femme allaitante
8. Ou tout autre critère apprécié par l'investigateur.

6.3 TRAITEMENTS PROHIBES

Les investigateurs restent libres de leur prise en charge habituelle pour leur patient atteint dediabète, mais tout traitement devra être documenté pendant l'étude, ainsi que les modifications éventuelles de traitement au cours de l'étude.
Il n'y a donc aucun traitement prohibé pendant l'étude.

6.4 CRITERES DE SORTIE PREMATUREE DU PATIENT DE L'ETUDE

Les patients pourront être sortis prématurément de l'étude dans les situations suivantes :

- Inclusion s'avérant non conforme au protocole
- Décision du patient : le patient est libre à tout moment de renoncer à participer à l'étude sans que cela ne porte préjudice à la qualité des soins ultérieurs
- Dysfonctionnement d'un dispositif médical ne permettant pas de continuer l'étude ou ne permettant pas de collecter les données avec fiabilité
- Toute raison clinique invoquée par l'investigateur
- Patient perdu de vue.

Si un patient sort prématurément de l'étude en raison d'un critère d'interruption spécifique, le motif et la date du retrait devront être rapportés dans le cahier d'observation.
Tout événement indésirable devra être déclaré et documenté selon les instructions détaillées figurant au chapitre 13.

6.5 REMPLACEMENTS

Les patients qui sortent d'étude ne seront a priori pas remplacés.

Si les abandons affectaient significativement l'effectif de patients dans l'étude, le Comité Scientifique serait sollicité pour prendre toute décision utile, comme des inclusions supplémentaires par exemple.

7 CALENDRIER DES VISITES ET PROCEDURES D'ETUDE

7.1 CALENDRIER DES VISITES

Pour cette étude, les visites ou contact suivants seront réalisés pour chaque patient par les investigateurs :

- Visite 1 (J0) : Visite d'Inclusion avec recueil du consentement éclairé et randomisation du type de pompe à utiliser
- Visite 2 (V2) 4 semaines après : Visite de Suivi après 4 semaines d'utilisation de la pompe
- Visite 3 (V3) 12 semaines après l'inclusion : Visite de Fin d'étude et mesure du critère principal de jugement (endpoint)
- Période d'un mois supplémentaire pour les patients du groupe « Omnipod » afin de leur permettre d'utiliser la pompe Medtrum (élément jugé facilitateur de la participation à l'étude pour les patients qui seraient randomisés dans le groupe comparateur)

7.2 CALENDRIER DES EVALUATIONS

Le calendrier des évaluations est présenté ci-après en ***Table 2***.

	screening	inclusion	1M	3M	1M supplémentaire
					patients du groupe Omnipod uniquement
recherche patients déjà utilisateurs Insulet + FreeStyle Libre	X				
s'assurer disponibilité HbA1c <1M et résultats glycémiques	X				
information du patient		X			
signature consentement		X			
critères d'inclusion et d'exclusion		X			
randomisation du type de pompe		X			
données socio-démographiques		X			
histoire de la maladie		X			
valeurs HbA1c et estimation A1c à l'inclusion		X			
événements glycémiques majeurs dernier mois et 6 derniers mois		X			
fourniture du matériel MEDTRUM		X			X
vérification du bon usage des dispositifs		X	X	X	
incidents éventuels avec les dispositifs			X	X	X
événements indésirables indésirables			X	X	X
recueil et report des données de glycémies et événements		X	X	X	X
recueil et report des données de la pompe			X	X	X
questionnaire de satisfaction de la pompe et du PDM		P	P	P	P
P = questionnaire renseigné par le patient					

Table 2 : Calendrier des évaluations

7.3 VISITE 1 : VISITE D'INCLUSION AVEC RECUEIL DU CONSENTEMENT ECLAIRE

Aucune procédure spécifique à l'étude ne doit être effectuée tant que le consentement éclairé du patient n'ait été obtenu.

Les procédures d'étude à la Visite 1 comprennent les évaluations suivantes :

- Fournir des informations verbalement sur les modalités et les objectifs de l'étude ainsi que celles présentées dans la notice d'information, et s'assurer de la possibilité pour le patient de poser des questions
- Obtenir la signature du consentement éclairé
- Recueillir les données suivantes à l'aide de l'eCRF :
- Données socio-démographiques
- Ancienneté du diabète, type, ancienneté de l'insulinothérapie
- HbA1C au moment de la prescription de la pompe à insuline
- Modèle actuel de pompe utilisée par le patient
- Satisfaction du patient de la pompe actuelle
- Type d'insuline utilisée
- Dernière valeur d'HbA1C datant de moins d'un mois
- Mesures du glucose disponibles (moyenne des mesures du glucose, HbA1c estimée, time in range)
- Nombre d'événements glycémiques majeurs au cours du dernier mois (tels que définis par l'ADA) et au cours des 6 derniers mois
- Randomisation du patient dans le groupe pompe « Omnipod » ou « Medtrum »

7.4 VISITE 2 (4 SEMAINES) : VISITE DE SUIVI APRES 4 SEMAINES D'UTILISATION DE LA POMPE

- Recueillir les données de tolérance et les éventuels événements indésirables, et procéder à la déclaration nécessaire depuis l'eCRF
- Vérifier les conditions d'utilisation des dispositifs par le patient
- Recueillir les enregistrements horodatés des valeurs de glucose (ainsi que l'HbA1c estimée à partir des mesures moyennes du glucose) et événements glycémiques, hypoglycémie et hyperglycémies, doses d'insuline administrées, à partir des logiciels fournis par les fabricants
- Recueillir les incidents techniques avec le dispositif (occlusion de cathéter, alarmes, décollement de la pompe, douleur, allergie, etc…)
- Satisfaction du patient de l'usage de son système d'administration de l'insuline
- Confirmer ou programmer la date de la visite V3 à 3 mois d'utilisation
- Enregistrer toutes les données pertinentes dans l'eCRF.

7.5 VISITE 3 (12 SEMAINES) : VISITE DE FIN D'ETUDE

- Recueillir les données de tolérance et les éventuels événements indésirables, et procéder à la déclaration nécessaire depuis l'eCRF
- Vérifier les conditions d'utilisation des dispositifs par le patient
- Recueillir les enregistrements horodatés des valeurs du glucose (ainsi que l'HbA1c estimée à partir des mesures moyennes du glucose) et événements glycémiques, hypoglycémies et hyperglycémies, doses d'insuline administrées

- Recueillir les incidents techniques avec le dispositif (occlusion de cathéter, alarmes, décollement de la pompe, douleur, allergie, etc…)
- Recueillir la satisfaction du patient et sa préférence pour l'un ou l'autre des pompes utilisées (pour les seuls patients du groupe Medtrum)
- Récupérer les matériels Medtrum
- Enregistrer toutes les données pertinentes dans l'eCRF
- Proposer au patient s'il est dans le groupe « Omnipod » de pouvoir utiliser une pompe Medtrum pendant un mois et prendre rendez-vous dans un mois pour une visite de fin d'étude et de récupération des matériels

7.6 VISITE 4 (4 SEMAINES APRES FIN D'ETUDE) : APRES 1 MOIS D'UTILISATION DE LA POMPE MEDTRUM (UNIQUEMENT PATIENTS RANDOMISES DANS LE GROUPE OMNIPOD)

- Recueillir les données de tolérance et les éventuels événements indésirables, et procéder à la déclaration nécessaire depuis l'eCRF
- Recueillir les enregistrements horodatés des valeurs du glucose (ainsi que l'HbA1c estimée à partir des mesures moyennes du glucose) et événements glycémiques, hypoglycémies et hyperglycémies, doses d'insuline administrées
- Recueillir les incidents techniques avec le dispositif (occlusion de cathéter, alarmes, décollement de la pompe, douleur, allergie, etc…)
- Recueillir la satisfaction du patient et sa préférence pour l'un ou l'autre des pompes utilisées
- Récupérer les matériels Medtrum
- Enregistrer toutes les données pertinentes dans l'eCRF

8 QUESTIONNAIRES PATIENT

Les patients seront avisés de remplir les questionnaires dans un endroit tranquille, et de ne pas être dérangés lors de leur remplissage. Il sera conseillé au patient de planifier le temps de remplissage des questionnaires nécessaires pour chaque visite. Le patient pourra renseigner les auto-questionnaires en consultation ou juste après avec l'aide de l'équipe soignante si besoin.

Les questionnaires qui devront être renseignés de façon contemporaine aux visites sont les suivants :

- Questionnaire de satisfaction sur l'utilisation de chaque pompe à insuline (à toutes les visites)

Il n'existe pas de questionnaire standardisé et validé en français permettant d'évaluer la satisfaction d'utilisation d'une pompe à insuline. Un questionnaire spécifique a donc été élaboré pour les besoins de cette étude.

Les investigateurs expliqueront aux patients l'importance de renseigner les auto-questionnaires, conformément aux instructions du protocole.

9 COLLECTE ET CONTROLE QUALITE DES DONNEES

9.1 SYSTEME D'INFORMATION

Une solution de gestion en ligne des observations cliniques de l'étude (eCRF) est mise à disposition des investigateurs de l'étude (Ennov Clinical).

Les données cliniques sont hébergées en France dans un data-center professionnel avec des garanties de sauvegarde et de protection conformes aux recommandations en recherche clinique. L'administration de la solution est gérée par Axonal-Biostatem et par Ennov.

Une plateforme web (cloud) est gérée par MEDTRUM pour permettre le recueil des données issues de la pompe. Cette plateforme est gérée par MEDTRUM, et une application permet aux investigateurs de visualiser les données et de faire des extractions à partir d'un site internet (cloud).

Une plateforme web (cloud) est utilisée pour collecter les données issues du lecteur de glycémie FreeStyle Libre de chaque patient. Cette plateforme est gérée par Abbott et une application permet aux investigateurs de visualiser les données et de faire des extractions.

Une plateforme web (cloud) Diasend® de Glooko est utilisée pour collecter les données issues de la pompe Omnipod®. Cette plateforme est gérée par Glookoet une application permet aux investigateurs de visualiser les données et de faire des extractions.

Aucune donnée nominative des patients ne sera recueillie dans l'eCRF, seuls les investigateurs connaitront l'identité des patients. Ni le promoteur ni le prestataire de service n'accèderont aux applications permettant de consulter les données des patients.

Les processus mis en œuvre seront conformes au Règlement Général européen sur la Protection des Données (RGPD). L'exercice des droits des investigateurs et des patients pourra se faire auprès de dataprotection@MEDTRUM.fr .

9.2 SAISIE DES DONNEES

9.2.1 Saisie par l'investigateur

Les données cliniques du patient seront recueillies par le médecin dans un eCRF accessible via internet. L'accès à l'eCRF sera individuel par login et mot de passe, et la finalisation du recueil des données nécessitera une signature électronique de l'investigateur.

Les modalités de saisie dans l'eCRF seront expliquées au médecin lors de la visite de mise en place de l'étude. Le médecin devra remplir l'eCRF le plus tôt possible après le recueil de l'information, de préférence le jour même de la visite.

Les médecins seront identifiés dans l'application par un numéro de centre.

Toutes les données des patients seront identifiées par un numéro unique spécifique à l'étude dans la base de données eCRF. Les patients seront également identifiés dans l'eCRF par leurs initiales (1ère lettre du prénom et 1ère lettre du nom de famille). Les investigateurs sont autorisés à détenir une liste de correspondance avec les données nominatives des patients, mais ces informations ne doivent pas figurer dans l'eCRF et ne doivent être communiquées à quiconque, en particulier ni au Promoteur ni au prestataire CRO.

9.2.2 Saisie par le patient

Les données des questionnaires de satisfaction renseignés par le patient (auto-questionnaires) seront recueillies sur papier et saisies par le centre investigateur dans l'eCRF.

9.3 CONTROLE QUALITE DES DONNEES

Le contrôle de la qualité de la base de données sera effectué tout au long de la conduite de l'étude afin de permettre un gel de la base de données finale dans des délais courts.

Les demandes de corrections seront générées automatiquement par le système. Les personnels de la CRO prestataire pourront générer manuellement depuis l'eCRF des demandes de corrections spécifiques si nécessaire.

Le centre logistique de l'étude (Axonal-Biostatem) veillera à communiquer régulièrement avec les investigateurs pour leur demander de répondre aux demandes de corrections sur l'eCRF demeurant non résolues.

Les médecins investigateurs seront consultés afin de recueillir leur accord pour toute correction de leurs données. Les données finales devront être contrôlées et approuvées par l'investigateur avec sa signature électronique.

9.4 GEL FINAL DE LA BASE DE DONNEES

Après que la base de données ait été déclarée « propre » pour analyse (c'est-à-dire la plus complète et précise), la base de données sera gelée et sauvegardée avant lancement des analyses statistiques, un certificat de gel sera documenté.

Toute modification de la base de données après cette date ne peut être faite que par un accord écrit conjoint entre le Promoteur de l'étude et le statisticien.

10 CRITERES D'EVALUATION ET DE SUIVI

10.1 CRITERE PRINCIPAL D'EVALUATION

Le critère principal d'évaluation est l'estimation de l'HbA1c à partir de la moyenne des mesures du glucose sur les 10 dernières semaines de suivi.

Les glycémies seront issues des mesures du capteur de glucose en continu FreeStyle Libre, extraites à partir de l'application fournie par le fabricant (déjà utilisée en routine) et reportées par l'investigateur dans l'eCRF. Il sera demandé à l'investigateur d'imprimer les mesures depuis le logiciel afin de faciliter le monitoring des données reportées.

Il n'y aura pas de recalcul de l'HbA1c estimée.

Nous avons fait le choix d'une évaluation de l'HbA1c obtenue à partir d'une mesure du capteur qui est une bonne estimation de la glycémie moyenne. L'évolution du taux classique d'HbA1c n'est pas informatif sur le temps passé en hyper ou en hypoglycémie (que ces dernières soient ou non symptomatiques) et ne reflète pas la variabilité glycémique. De plus si l'étude de Nathan[17] souligne la fiabilité de l'estimation de l'HbA1C sur 14 jours de mesures continues avec un capteur, tout comme, en 2017, les Recommendations ADA [25] ;

plus recemment celles de l'« International Consensus on Time in Range »[26] publiées en 2019 rappellent les limites du critère HbA1c au laboratoire. L'évolution du suivi de l'efficacité du traitement insulinique s'oriente de plus en plus vers la glycémie moyenne et sur le « Time In Range » (temps passé dans l'objectif fixé /les valeurs cibles).

Nous proposons d'évaluer, en critère secondaire, l'évolution de l'HbA1c mesurée au laboratoire à partir d'un prélèvement sanguin en début et fin d'étude, ainsi que le Time in Range qui est calculé automatiquement à partir des données issues du lecteur FreeStyle Libre.

10.2 CRITERES SECONDAIRES D'EVALUATION

Les critères secondaires d'évaluation sont les suivants :

- **HbA1c :**
  - Valeur mesurée au laboratoire à partir d'un prélèvement sanguin réalisé avant la visite J0 et juste avant la visite à 3 mois

- **Mesures du Taux de glucose**
  - Moyenne des Mesures du glucose sur chaque période et sur la durée totale de l'étude
  - Valeurs minimums et maximum
  - Temps passé dans la cible (« time in range ») exprimé en % (cf Recommendations ADA et celles de l'« International Consensus on Time in Range »[25,26])

  Ces valeurs sont calculées automatiquement par le logiciel du FreeStyle Libre et seront reportées par l'investigateur.

- **Evénements glycémiques**
  - Nombre d'hypoglycémies, symptomatiques ou non sur la période d'évaluation, réparties selon le niveau de gravité (cf infra)
  - Nombre d'hyperglycémies, symptomatiques ou non sur la période d'évaluation, réparties selon le niveau de gravité (cf infra)

  Ces informations sont issues des données du FreeStyle Libre et des données renseignées par le patient dans le PDM.

  La définition des hypoglycémies et hyperglycémies est celle du consensus de l'ADA[25] :

Table 1—Summary of consensus definitions

Outcome	Definition
Hypoglycemia	Level 1: glucose $<$70 mg/dL (3.9 mmol/L) and glucose $\geq$54 mg/dL (3.0 mmol/L)<br>Level 2: glucose $<$54 mg/dL (3.0 mmol/L)<br>Level 3: a severe event characterized by altered mental and/or physical status requiring assistance
Hyperglycemia	Level 1—elevated glucose: glucose $>$180 mg/dL (10 mmol/L) and glucose $\leq$250 mg/dL (13.9 mmol/L)<br>Level 2—very elevated glucose: glucose $>$250 mg/dL (13.9 mmol/L)
Time in range	Percentage of readings in the range of 70–180 mg/dL (3.9–10.0 mmol/L) per unit of time

- **Tolérance des dispositifs**
    - Tolérance locale au niveau de la peau à l'emplacement de la pompe (maintien par adhésif) et à l'emplacement du capteur de glycémie FreeStyle Libre (maintien par adhésif).
    - Tout problème de tolérance locale ou générale devra être rapporté pendant l'étude, y compris dans la phase d'un mois supplémentaire en fin d'étude pour les patients du groupe « Omnipod »

- **Incidents avec les dispositifs**
    - Tout type d'incident de fonctionnement des dispositifs médicaux utilisés, en particulier pour les pompes : arrachement, problème de commande, difficulté de transmission, obstruction, fuite, etc

- **Satisfaction des patients**
    - Auto-questionnaire évaluant la satisfaction d'utilisation du dispositif (pompe+PDM)
    - Question finale portant sur la préférence du patient entre le dispositif Medtrum et le dispositif Insulet

- **Traitement par insuline**
    - Le traitement par insuline utilisé devra être documenté
    - Les doses d'insuline délivrées seront issues de l'enregistrement des données de la pompe
    - Bien que non autorisées dans l'étude, les éventuelles injections complémentaires d'insuline à l'aide d'un stylo seront documentées le cas échéant

10.3 AUTRES CRITERES

- Non applicable.

11 ANALYSE STATISTIQUE

11.1 JUSTIFICATION DU NOMBRE NECESSAIRE DE PATIENTS

L'objectif d'une étude de non-infériorité est de démontrer que la différence moyenne entre les 2 dispositifs demeure faible et cliniquement non significative.

La glycémie moyenne obtenue dans la vie réelle avec la pompe Omnipod est de 7,8% selon l'expérience des trois centres du comité scientifique de l'étude, ce qui est conforme aux données de la littérature. Nous pouvons supposer que la glycémie moyenne sous La pompe Medtrum est la même que sous la pompe Omnipod.

En fixant la borne Δ de non infériorité à +0.4%, conformément aux recommandations de la FDA (Guidance for Industry Diabetes Mellitus (Developing Drugs and Therapeutic Biologics for Treatment and Prevention)), si la borne supérieure de l'IC95% de la différence entre les 2 pompes (Medtrum-Omnipod) dépasse Δ (soit 0.4), la non infériorité ne sera pas démontrée.

Dans le cas contraire, la supériorité de la pompe Medtrum sur la pompe Omnipod pourra être testée, avec une borne Δ’ fixée à -0.3%.

Sur la base de ces hypothèses, basé sur le design dit du « Less is better » avec α = 2,5%, β = 20% et un écart-type (ET) fixé à 0,55, le calcul du nombre nécessaire de sujets (analysables) est de 60 patients (30 dans chaque groupe).

En supposant que 20 % des patients ne peuvent être analysés (écarts majeurs, données manquantes, perdus de vue), le nombre de patients à randomiser est de 75. Le nombre de centres à recruter en 3 mois maximum est de 6.

Pour confirmer notre hypothèse sur l'écart-type, une analyse descriptive des données de base d’HbA1c pour l'ensemble de la population sera effectuée en aveugle à la fin de la période d'inclusion. Si une forte différence par rapport à nos hypothèses est trouvée, la taille de l'échantillon sera calculée de nouveau, afin de conserver une puissance statistique de 80 % pour cette étude.

11.2 ANALYSE STATISTIQUE

11.2.1 Méthodes statistiques générales

Le traitement des données et les analyses statistiques seront réalisés par la société prestataire, Axonal-Biostatem.

Les analyses statistiques seront décrites dans un plan d’analyse statistique (PAS) validé par le Promoteur et le Comité Scientifique, et ce avant le gel de base.

Les analyses statistiques seront réalisées après le gel de base des données avec le logiciel SAS® (SAS Institute, NC, Cary, USA), version 9.4 ou ultérieure.

Les analyses des données démographiques seront effectuées dans la ITT. Les analyses de tolérance seront faites dans la population de tolérance. Les analyses de Non-infériorité seront réalisées sur la population Per Protocol puis validées sur la population ITT.

Pour les variables quantitatives, les statistiques usuelles (n, n manquants, moyenne, écart type (ET), médiane, premier et troisième quartiles (Q1 and Q3), minimum et maximum) seront présentées. Les IC95% pourront être présentés si pertinent (notamment sur le critère de jugement principal).

Pour les variables qualitatives, les statistiques usuelles (n, n manquants, fréquence et pourcentage) de chaque modalité seront fournies.

Les statistiques descriptives seront fournies au global.

L’erreur de type 1,α, est fixée à 5% pour toutes les analyses.

11.2.2 Populations étudiées

Pour répondre aux objectifs de l’étude, 2 populations seront définies :

- ITT : tous les patients inclus, ayant utilisé au moins une fois le dispositif de pompe. Les patients seront analysés selon leur groupe alloué par la randomisation.
- Per Protocol : Patients de l’ITT ne présentant aucune déviation majeure au protocole.

- Population de tolérance : tous les patients ayant utilisé le dispositif de pompe au moins une fois.

11.2.3 Analyse Descriptive

Toutes les variables recueillies feront l'objet d'une description et/ou d'un listing de données individuelles.

Les variables d'efficacité et de tolérance seront analysées selon les sections ci-dessous (11.2.4 et 11.2.5). Les caractéristiques socio-démographiques, cliniques, les antécédents médicaux ainsi que les traitements seront décrits selon la méthodologie présentées en section 11.2.1.

11.2.4 Analyse de l'efficacité/performance du dispositif

Analyse du critère principal d'efficacité :

L'analyse de Non infériorité du critère principal d'efficacité sera réalisée avec une ANOVA, avec le DM en variable explicative, sur la population *per protocol* puis sur la population *en intention de traiter*. Si la borne supérieure de l'IC95% de la différence de moyenne estimée (LSmeans) Omnipod-Medtrum dépasse la borne de non infériorité, celle-ci sera rejetée.

Les bornes de non-infériorité sont définies dans ce protocole et seront utilisées dans le plan d'analyse statistique.

Dans le cas où la non-infériorité est démontrée, une analyse de supériorité sera conduite.

Analyses des critères secondaires d'efficacité :

L'analyse des critères secondaires d'efficacité portera sur les variables listées ci-dessous.

Chaque variable sera décrite globalement et par groupe de pompe.

En cas d'analyse de non infériorité, la même méthodologie que pour le critère de jugement principale sera effectuée.

En cas de comparaison de variables quantitatives, des tests de comparaison de moyennes paramétriques (du type test T) ou non paramétriques (de type Mann et Whitney) seront utilisés. En cas de comparaison de variables qualitatives, des tests du CHI² ou du Fisher exact seront utilisés.

En cas de tests sur des évolutions au sein de chaque groupe, des tests sur données appariées seront utilisés (Test T sur données appariées ou test des rangs signés de Wilcoxon, selon la distribution des données).

- **HbA1c :**
  - Evolution de la valeur mesurée au laboratoire à partir d'un prélèvement sanguin réalisé avant la visite J0 et juste avant la visite à 3 mois
  - Description dans la population totale, dans chaque groupe et comparaison entre les groupes.

- **Mesures du taux de glucose**
  - Moyenne des mesurs du glucose sur chaque période et sur la durée totale de l'étude
  - Description des valeurs minimum et maximum sur la durée totale de l'étude
  - Description du % de Time in range (cf Recommendations ADA et celles de l'« International Consensus on Time in Range »[25,26])
  - Description dans la population totale, dans chaque groupe et comparaison entre les groupes.

- **Evénements glycémiques**
  - Description du nombre d'hypoglycémies, symptomatiques ou non sur la période d'évaluation, réparties selon le niveau de gravité (cf définitions du consensus de l'ADA[25])
  - Description du nombre d'hyperglycémies, symptomatiques ou non sur la période d'évaluation, réparties selon le niveau de gravité (cf définitions du consensus de l'ADA[25])
  - Description dans la population totale, dans chaque groupe et comparaison entre les groupes.
  -

- **Tolérance des dispositifs**
  - Description de la tolérance locale au niveau de la peau à l'emplacement de la pompe et à l'emplacement du capteur de glycémie FreeStyle Libre).
  - Description de tout problème de tolérance locale ou générale rapporté pendant l'étude, y compris dans la phase d'un mois supplémentaire en fin d'étude pour les patients du groupe « Omnipod »
  - Description dans la population totale, dans chaque groupe et comparaison entre les groupes du nombre de patients ayant eu au moins un problème de tolérance.

- **Incidents avec les dispositifs**
  - Description de tout type d'incident de fonctionnement des dispositifs médicaux utilisés, en particulier pour les pompes : arrachement, problème de commande, difficulté de transmission, obstruction, fuite, etc
  - Description dans chaque groupe, dans la population globale et comparaison entre les groupes

- **Satisfaction des patients**
  - Description des réponses à l'auto-questionnaire évaluant la satisfaction d'utilisation du dispositif (pompe+PDM), dans chaque groupe et dans la population totale
  - Comparaison des réponses entre les groupes
  - Description des réponses à la question finale portant sur la préférence du patient entre le dispositif Medtrum et le dispositif Omnipod : dans chaque groupe et dans la population totale

- **Traitement par insuline**
  - Description des doses d'insuline délivrées

- Description des éventuelles injections complémentaires d'insuline à l'aide d'un stylo pendant l'étude
- Description dans la population totale, dans chaque groupe et comparaison entre les groupes

11.2.5 Analyses de la tolérance :

Les analyses seront effectuées sur la population de tolérance.

Avant toute analyse tous les incidents liés au DM et la tolérance liée au DM seront codés par le dictionnaire médical réglementaire MedDRA (version 20.0 ou ultérieure).

Une table résumée (nombre et pourcentage de patients ayant un EI et nombre d'événements) par classe de système d'organes (SOC) et terme préférentiel (Preferred Term, PT) sera présentée pour les catégories suivantes d'événements en dissociant incidents et tolérance liés au DM :

- Selon la liaison (dispositif ET procédure),
- Selon la liaison (dispositif OU procédure),
- Selon la gravité,
- Selon les actions prises (désapareillage).

Un listing de données individuelles sera fourni décrivant toutes les informations relevées via l'eCRF concernant les EIs (notamment durée, résolution, critère de gravité, liaison, sévérité etc.)

11.2.6 Analyse intermédiaire

Aucune analyse intermédiaire n'est prévue.

En revanche, une analyse descriptive des données de base de l'HbA1c pour l'ensemble de la population sera effectuée en aveugle à la fin de la période d'inclusion, afin de confirmer ou non l'hypothèse sur l'écart-type prévue pour l'analyse de non-infériorité. Cette analyse étant purement descriptive aucun ajustement du risque α n'est prévu.

11.2.7 Analyses en sous-groupes, analyses exploratoires

Elles seront éventuellement décrites dans le plan d'analyse statistique final.

12 CONTROLES QUALITE

Monitoring

Cette étude sera suivie à toutes les étapes de sa réalisation par des ARCs de la Société prestataire (AXONAL-BIOSTATEM) mandatée par le Promoteur.

Cette étude clinique sera conduite selon les bonnes pratiques de la norme ISO 14155 et si applicable selon les recommandations ICH-GCP E6(R2).

Les ARC du prestataire seront chargés de la mise en place, du suivi, du monitoring et des clôtures des centres.

Lors de la mise en place de l'étude, l'ARC fournira une formation sur le contenu du protocole et sur l'eCRF aux médecins participants. Une formation des centres portant sur l'utilisation du dispositif médical sera effectuée par un représentant habilité du Promoteur.

A la demande du Promoteur, des visites sur site pourront être également effectuées régulièrement selon un calendrier déterminé. Lors de ces visites, l'investigateur permettra à l'ARC d'accéder directement aux différents documents de l'étude : cahier d'observation, consentement éclairé, classeur investigateur et documents sources, et ce dans le respect de la confidentialité. Durant ces visites, l'ARC contrôlera les formulaires d'information et de consentement du patient, pourra comparer les données inscrites dans les eCRFs avec les données source (données manquantes, aberrantes), et s'assurera que l'étude est réalisée dans le respect du protocole et des Bonnes Pratiques Cliniques (BPC).

L'ARC vérifiera également, à chaque visite, que tous les événements indésirables (EI) et en particulier les événements indésirables graves (EIGs) éventuellement observés au cours de l'étude, sont notifiés dans les délais prévus.

A la fin de l'étude une visite de clôture sera réalisée, et l'ARC se chargera du retour des dispositifs médicaux utilisés/non utilisés auprès du Promoteur.

<u>Audits/Inspections</u>

A la demande du Promoteur, des audits peuvent être effectués pour vérifier la qualité des données, leur authenticité et le respect des procédures mentionnées dans le protocole. Le cas échéant, ces audits seront effectués au cours de l'étude et/ou à la fin de l'étude par des auditeurs indépendants de l'équipe responsable de la mise en œuvre et du suivi de l'étude.

Par ailleurs, les représentants des autorités de santé françaises, ainsi que ceux du Comité de Protection des Personnes sont susceptibles d'inspecter les centres investigateurs à tout moment. L'investigateur ayant connaissance de la réalisation d'un tel audit devra informer immédiatement le promoteur.

L'investigateur devra s'assurer de sa disponibilité le jour de l'audit/inspection et garantir le libre accès des auditeurs à tous les documents sources.

13 EFFETS INDESIRABLES, VIGILANCE

Les responsabilités de chaque intervenant en matière de vigilance seront détaillées dans un document (Safety management Plan) validé par le promoteur.

La CRO (Axonal-Biostatem) sera notamment en charge du recueil des incidents liés au DM et des incidents de tolérance liée au DM au correspondant vigilance de l'étude (VIGIPHARM).

Le Promoteur se chargera des demandes d'informations complémentaires aux investigateurs, de la rédaction de narratifs, de la soumission des rapports des incidents liés au DM et des incidents de tolérance liée au DM auprès des investigateurs et du CPP et de la réconciliation des données provenant de la base de données de vigilance et la base de données cliniques.

Le promoteur s'assurera d'identifier les incidents liés au DM et les incidents de tolérance liée au DM, de maintenir une base de données pour la déclaration des incidents liés au DM et des incidents de tolérance liée au DM, de déclarer les incidents liés au DM et les incidents de tolérance liée au DM aux autorités compétentes et de préparer les rapports concernant la vigilance.

13.1 DEFINITIONS

Un évènement indésirable (EI) est tout événement médical indésirable, maladie ou blessure involontaire ou signes cliniques indésirables (y compris anomalie, résultats de laboratoire) chez des sujets, des utilisateurs ou d'autres personnes, qu'ils soient ou non liés au traitement médical expérimental. Cette définition inclut les événements liés au dispositif médical expérimental ou au comparateur et les événements liés aux procédures impliquées. Pour les utilisateurs ou autres personnes, cette définition est limitée aux événements liés aux dispositifs médicaux de recherche.

Effet indésirable d'un dispositif médical (Adverse Device Effect ADE) : Evenement indésirable lié à l'utilisation du dispositif médical sous investigation. Cette définition inclut tout événement indésirable résultant d'insuffisances ou d'inadéquations dans les instructions d'utilisation, le déploiement, l'implantation, l'installation et le fonctionnement, ou tout dysfonctionnement du dispositif médical sous investigation. Elle inclut tout événement résultant d'une erreur d'utilisation ou d'un usage impropre intentionnel du dispositif médical sous investigation

Effet indésirable grave du dispositif (Serious Adverse Device Effect SADE)

Est un effet indésirable du dispositif entraînant l'une des conséquences caractéristiques d'un événement indésirable grave décrite ci-dessous

Un évènement indésirable Grave (EIG) est tout EI qui :

- Entraîne la mort ou
- Entraîne de graves détériorations de l'état de santé du sujet, ce qui a eu pour conséquence
  - la mise en danger de la vie, une maladie ou une blessure ou,
  - une déficience permanente d'une structure ou d'une fonction corporelle ou,
  - une hospitalisation ou la prolongation d'une hospitalisation ou,
  - une intervention médicale ou chirurgicale afin de prévenir la mise en danger de la vie, une maladie, une blessure ou une déficience permanente d'une structure ou d'une fonction du corporelle
- Entraîne une détresse fœtale, la mort fœtale, une anomalie congénitale ou une malformation congénitale.

Remarque : une hospitalisation planifiée pour une condition préexistante, ou une procédure requise par le protocole, sans gravité sur la détérioration de l'état de santé, ne sont pas considérés comme un événement indésirable grave.

Le jugement médical et scientifique doit permettre de décider si d'autres EIs doivent aussi être considérés comme graves, tels que des évènements médicaux pertinents qui peuvent ne

pas mettre immédiatement la vie en danger, contribuer au décès ou provoquer une hospitalisation mais qui peuvent constituer un danger potentiel pour le patient ou nécessiter une intervention pour prévenir l'une des conséquences listées dans la définition ci-dessus

Un effet indésirable inattendu : tout effet indésirable dont la nature, la sévérité ou l'évolution ne concorde pas avec les informations relatives aux produits, actes pratiqués et méthodes utilisées au cours de la recherche

Suspicion d'effets indésirables : Tous les EIs pour lesquels l'investigateur ou le promoteur estiment qu'une relation de causalité avec l'élément expérimental peut être raisonnablement envisagée sont considérés comme des suspicions d'effets indésirables.

Suspicions d'effets indésirables graves inattendus (EIGI ou SUSAR) : Une suspicion d'effet indésirable grave inattendu correspond à toute réaction nocive et non désirée à un produit expérimental, quelle que soit la dose administrée :

- Qui entraîne la mort, met en danger la vie de la personne qui se prête à la recherche, nécessite une hospitalisation ou la prolongation de l'hospitalisation, provoque une incapacité ou un handicap important ou durable, ou bien se traduit par une anomalie ou une malformation congénitale ;
- Dont la nature, la sévérité, la fréquence ou l'évolution ne concorde pas avec les informations relatives aux produits, actes pratiqués et méthodes utilisées au cours de la recherche ;
- Pour laquelle l'investigateur ou le promoteur estime qu'une relation de causalité avec le dispositif médical expérimental peut être raisonnablement envisagée.

Fait nouveau : Toute nouvelle donnée pouvant conduire à une réévaluation du rapport des bénéfices et des risques de la recherche ou du produit objet de la recherche, à des modifications dans l'utilisation de ce produit, dans la conduite de la recherche, ou des documents relatifs à la recherche, ou à suspendre ou interrompre ou modifier le protocole de la recherche ou des recherches similaires.

Intensité : L'intensité des événements indésirables est évaluée par l'investigateur en s'aidant de la classification suivante :

- Léger de grade 1 : événement indésirable généralement transitoire et sans retentissement sur les activités normales ;
- Modéré de grade 2 : événement indésirable suffisamment gênant pour retentir sur les activités normales ;
- Sévère de grade 3 : événement indésirable modifiant considérablement le cours normal des activités du patient, ou invalidant, ou constituant une menace pour la vie du patient.

Défectuosité du dispositif

- Insuffisance du dispositif médical liée à son identité, sa qualité, sa durabilité, sa fiabilité, sa sécurité ou ses performances

Remarque : Les défectuosités du dispositif incluent des dysfonctionnements, des erreurs d'utilisation et un étiquetage inadéquat.

Toutes les défectuosités du dispositif médical liés à son identité, sa qualité, sa durabilité, sa fiabilité, sa sécurité ou ses performances doivent être documentées tout au long de l'essai clinique et correctement gérées par le sponsor.

Les défectuosités du dispositif médical qui n'ont pas entraîné d'effet indésirable, mais auraient pu entraîner un événement médical

a) si aucune des mesures appropriées n'avait été prise,

b) si l'intervention n'avait pas été faite, ou

c) si les circonstances avaient été moins chanceuses,

doivent être signalées dans le cadre de l'étude

Imputabilité : L'investigateur et le promoteur évaluent à la fois la relation de l'événement indésirable (EI) avec le dispositif sous investigation et la relation de l'EI avec une intervention prévue par le protocole

13.2 Responsabilites de l'investigateur

13.2.1 Notification des évènements indésirables (EI)

Les patients seront encouragés à signaler tous incidents liés au DM et incidents de tolérance liée au DM et les rapporter à l'investigateur.

À chaque évaluation, l'investigateur mènera un interrogatoire auprès du patient pour déterminer si des effets indésirables se sont produits.

Le médecin rapportera dans l'eCRF tous incidents liés au DM et incidents de tolérance liée au DM observé ou rapporté spontanément par le patient pendant toute la durée de l'étude (dès l'obtention du consentement signé du patient pour participer à l'étude).

Les EIs non réliés au DM ou non reliés à la procédure d'implantation du DM ne seront pas renseignés dans l'eCRF. Toutefois, les médecins se devront de déclarer tout effet indésirable suspecté d'être dû à un médicament ou produit par courrier au centre régional de pharmacovigilance (CRPV) dont il dépend ou sur le site http://solidarites-sante.gouv.fr/soins-et-maladies/signalement-sante-gouv-fr.

Les incidents liés au DM et les incidents de tolérance liée au DM seront documentés en détails dans le cahier d'observation (eCRF). Les informations suivantes devront être renseignées :

- Date et heure d'apparition de l'EI,
- Durée de l'EI (indication de la durée totale de l'EI ou du symptôme ou détermination de cette durée à partir des dates et heures de début et de fin d'évolution),
- Intensité de l'EI (légère, modérée, sévère),
- La relation de causalité de l'EI avec le DM ET/OU avec la procédure liée au DM,
- Toute action entreprise vis-à-vis du produit à l'étude et pour traiter cet EI,
- Evolution de l'EI (ex. résolution complète, persistance…),

L'investigateur devra évaluer l'intensité, la gravité et la causalité de l'ensemble des évènements indésirables.

Ces derniers devront être suivis jusqu'à résolution de l'évènement.

Détermination de la durée :

Si la durée totale d'évolution de l'EI ou du symptôme n'est pas directement indiquée, elle sera calculée à partir de sa date d'apparition et de sa date de fin.

Appréciation de l'intensité :

L'investigateur précisera l'intensité de l'EI selon la classification suivante : légère, modérée, sévère en prenant en compte les degrés d'intensité possibles de l'évènement selon les définitions suivantes :

Léger	▪ Entraine un inconfort léger ou passager, n'exigeant pas d'intervention ou de traitement.<br>▪ Ne limite pas ou n'interfère pas dans les activités quotidiennes.
Modéré	▪ Entraine suffisamment d'inconfort pour limiter ou interférer avec les activités quotidiennes.<br>▪ Peut nécessiter un traitement.
Sévère	▪ Entraine des symptômes importants qui empêchent les activités quotidiennes normales.<br>▪ Peut nécessiter une intervention invasive.

Imputabilité / relation causale avec le produit de l'étude et la recherche :

Le promoteur se prononcera sur le lien de causalité de l'évènement survenu.

Relié	Tout évènement clinique ou biologique avec une relation chronologique et sémiologique compatible avec la survenue de l'EI.
Non relié	L'EI est clairement relié à d'autres causes, comme l'état clinique du patient ou un traitement concomitant.

Appréciation de l'évolution de l'EI :

L'investigateur informera également le Promoteur de toute nouvelle information relative au suivi.

L'investigateur précisera l'évolution de l'EI selon la classification suivante :

- Evolution favorable/guérison,
- En cours de guérison,
- Persistance de l'EI,
- Résolution / guérison avec effets résiduels (à préciser),
- Décès,
- Evolution inconnue.

En fin d'étude, un listing des incidents liés au DM et incidents de tolérance liée au DM non graves sera édité.

13.2.2 Notification des Evénements indésirables graves (EIG)

<u>L'investigateur est responsable de notifier au promoteur via Axonal-Biostatem **sans délai** à compter du jour où il en a connaissance tous les évènements graves liés au DM survenus au cours de la recherche.</u>

En cas d'EIG, l'investigateur complètera le formulaire spécifique pour les EIG fourni par le Promoteur. Tout EIG lié au DM devra néanmoins être également reporté sur les pages de l'eCRF destinées au recueil de la vigilance.

Dès que l'investigateur aura connaissance de la survenue d'un EIG lié au DM, il doit notifier i**mmédiatement au plus tard dans les 24 h après avoir eu connaissance de l'évènement** par e-mail **le Promoteur, ou ses représentants ainsi que la CRO en charge de la logistique de l'étude** (Axonal-Biostatem), via l'envoi du formulaire d'EIG dûment rempli au :

Correspondant vigilance :

Responsable du service vigilance :

CAROLINE NAVARRE

VIGIPHARM

265 rue Maurice Béjart

34 080 Montpellier

Tel: + 33 467 107 252- Fax : +33 (0)4-67-10-72-53

medtrum@vigipharm.fr

Ce formulaire d'EIG doit être complété et envoyé pour tous les EIG, indépendamment d'une relation causale possible. Ce formulaire est également accessible dans l'eCRF.

Il est demandé à l'investigateur de documenter en détail dans le rapport d'EIG le déroulement de l'EI, ainsi que tout traitement administré et toute donnée pertinente. L'investigateur devra se prononcer sur la causalité de l'évènement par rapport à la recherche sur le formulaire d'EIG.

L'investigateur doit suivre l'EIG jusqu'à sa résolution et informer le Promoteur de toute nouvelle information relative au suivi, ainsi que de l'évolution de l'EIG (rapport de suivi).

Dans le cas d'un décès, il est demandé à l'investigateur d'adresser au Promoteur toute information complémentaire disponible (ex. : rapport d'autopsie, compte rendu médical).

La CRO informera immédiatement le correspondant vigilance de l'étude (VIGIPHARM) en leur transmettant le formulaire d'EIG, et en s'assurant de leur correcte transmission.

13.2.3 Notification des grossesses

L'investigateur devra également notifier au correspondant de vigilance de la CRO (Axonal-Biostatem) tout cas de grossesse.

La CRO informera immédiatement le correspondant vigilance de l'étude (VIGIPHARM) en leur transmettant le formulaire de déclaration des grosseses, et en s'assurant de leur correcte transmission.

En cas de notification de cas grossesse, un suivi de l'EI sera réalisé jusqu'à 3 mois après la naissance de l'enfant. Par ailleurs, si la naissance est programmée après le gel de base, les informations reçues seront traitées postérieurement.

13.2.4 Notification des surdosages

L'investigateur devra également notifier au correspondant de vigilance de la CRO (Axonal-Biostatem) tout cas de surdosage de médicament.

13.2.5 Notification des Faits nouveaux

Tout fait nouveau pouvant être suffisant pour envisager des modifications dans l'utilisation du dispositif médical testé, dans la conduite de la recherche ou des documents relatifs à la recherche, ou qui pourrait conduire le cas échéant à une réévaluation des bénéfices et des risques de la recherche devra être rapporté au Promoteur **sans délai** par l'investigateur.

13.2.6 Notification des incidents de vigilance

Tous les incidents de vigilance devront être signalés par l'investigateur après en avoir pris connaissance par e-mail au Promoteur, ou à ses représentants et à la CRO en charge de la logistique de l'étude (Axonal-Biostatem), via l'envoi du formulaire de vigilance dûment rempli.

Correspondant vigilance :

**Responsable du service vigilance :**
**CAROLINE NAVARRE**
**VIGIPHARM**
**265 rue Maurice Béjart**
**34 080 Montpellier**
**Tel: + 33 467 107 252- Fax : +33 (0)4-67-10-72-53**
**medtrum@vigipharm.fr**

Notification sans délai

La notification des incidents ou des risques d'incident grave mettant en cause un dispositif médical ayant entrainé ou susceptible d'entraîner la mort ou la dégradation grave de l'état de santé d'un patient devra être réalisée sans délai :

- Décès du patient ou menace du pronostic vital,
- Invalidité ou incapacité permanente ou importante,
- Nécessité d'hospitalisation ou prolongation d'hospitalisation,
- Toute circonstance nécessitant une intervention médicale ou chirurgicale
- Survenue d'une anomalie ou malformation congénitale.

Autres incidents

Tous les autres incidents suivants devront également être notifiés par l'investigateur :

- Tout dysfonctionnement ou toute altération des caractéristiques ou des performances du dispositif médical,
- Toute réaction nocive et non voulue se produisant lors de l'utilisation du dispositif médical conformément à sa destination,

- Toute réaction nocive et non voulue résultant d'une utilisation d'un dispositif médical ne respectant pas les instructions du fabricant.

13.3 Responsabilites du promoteur

Le Promoteur est responsable de l'évaluation en continu de la sécurité de la recherche et de la transmission des informations en matière de vigilance aux autorités réglementaires compétentes.

Le promoteur transmettra à tous les investigateurs concernés via Axonal-Biostatem les informations susceptibles d'affecter la sécurité des personnes se prêtant à la recherche, notamment toute information pertinente relative aux suspicions d'EIGI liés au DM ou tout fait significatif relatif à la sécurité.

13.4 Comite de surveillance

Tous les incidents concernant le dispositif médical seront recueillis pendant l'étude et évalués par un Comité de surveillance.

Ce Comité de surveillance sera constitué de 3 membres indépendants.

Chaque membre devra signer une attestation d'absence de conflit d'intérêt pécuniaire potentiel en rapport avec les résultats de l'étude, et ne devra pas être connu pour avoir une « opinion tranchée » sur les mérites relatifs des interventions testées dans l'étude.

Ce Comité se réunira à la requête du Comité Scientifique, du Promoteur ou des investigateurs.

Il aura pour responsabilité de :

- Réaliser une revue régulière des éléments de sécurité et de performance de l'étude pour assurer la sécurité des participants, et que le rapport bénéfice / risque reste favorable à la poursuite de l'essai
- Réaliser la veille concernant les informations médicales ou scientifiques rendues publiques qui peuvent avoir un impact sur l'étude en cours et la sécurité des participants
- Réaliser l'adjudication des EIG pour statuer sur l'imputabilité ou non du dispositif médical et/ou de la procédure d'utilisation
- Rédiger des recommandations sur la continuation, la modification ou l'arrêt de l'étude à l'attention du Comité Scientifique et du Promoteur, d'après les critères ci-dessous.

Un arrêt de l'étude est envisagé si :

- Les données montrent une augmentation statistiquement significative du risque d'évènements indésirables rendant le rapport bénéfice/risque inacceptable,
- Un risque sérieux et inattendu est détecté,
- L'état de l'art rend la technologie étudiée obsolète.

14 CONSIDERATIONS ETHIQUES ET LEGALES

14.1 CADRE REGLEMENTAIRE DE L'ETUDE

Cette étude sera menée dans le respect des principes éthiques de la Déclaration d'Helsinki révisée en 2013, des Bonnes Pratiques Cliniques ICH-GCP E6(R2), de la norme ISO 14155, du Règlement Européen sur les dispositifs médicaux 2017/745 et de la législation française relative aux études cliniques.

Selon la législation française actuelle, cette étude est considérée comme une recherche impliquant la personne humaine (RIPH) de type II (étude interventionnelle à risques et contraintes minimes concernant un dispositif médical marqué CE non commercialisé en France).

14.2 SOUMISSION DU PROTOCOLE ET DU CONTRAT D'ETUDE

L'étude ne débutera qu'après obtention d'un avis favorable du Comité de Protection des Personnes tiré au sort et sera envoyé pour information auprès de l'ANSM.

14.2.1 Déclaration aux Autorités Réglementaires Compétentes

L'étude a été enregistrée dans la base française et dans la base européenne des études sous le N° ID-RCB : 2019-A02566-51.

Ce protocole a été envoyé pour information à l'Agence Nationale de Sécurité des Médicaments et des produits de santé (ANSM) en France.

Tout amendement (correspondant à des modifications substantielles) sera envoyé pour information à destination de l'Autorité Compétente avant sa mise en application.

Tout amendement du protocole devra être transmis par écrit à tous les investigateurs et être signé et daté par le promoteur et les investigateurs.

14.2.2 Déclaration au Comité d'éthique

Le protocole a été soumis en France au Comité de Protection des Personnes (CPP) SUD-EST VI.

Ni l'investigateur, ni le promoteur ne pourront modifier ce protocole sans que cette modification n'ait préalablement fait l'objet d'un accord écrit entre les deux parties. Toute modification considérée comme significative par l'investigateur ou le responsable de l'étude, devra être approuvée par le CPP avant sa mise en application.

14.2.3 Protection des données à caractère personnel

L'étude sera réalisée en conformité avec le Règlement Général européen de Protection des Données (RGPD) et avec la législation française (CNIL).

14.2.4 Déclaration aux Ordres Professionnels

L'étude sera en conformité avec les obligations ordinales, et respectera les obligations de transparence des liens d'intérêt.

Les données concernant les patients et les médecins seront recueillis en application du chapitre IX de la loi 78-17 du 6 janvier 1978 modifiée par la loi 2004-801 du 6 août 2004 (dite "Loi Informatique et Libertés") et de l'article 16 de la loi n° 2018-493 du 20 juin 2018 relative à la protection des données personnelles.

Ce protocole de Recherche Impliquant la Personne Humaine sera réalisé conformément à la méthodologie de référence MR-001 de la CNIL du 21 juillet 2016 (Commission Nationale Informatique et Libertés).

Un dossier de compliance à la MR-001 a été constitué afin de documenter le cadre applicable de l'étude. Le Promoteur a réalisé au préalable les démarches auprès de la CNIL en s'engageant sur le respect des méthodologies de référence.

14.3 INFORMATION ET CONSENTEMENT DU PATIENT

Les patients auprès desquels sont recueillies les données à caractère personnel seront individuellement informés par les médecins investigateurs avant le début de l'étude (c'est à dire avant tout examen éventuellement nécessaire pour le sélectionner dans l'étude) des objectifs de l'étude, des modalités, de la nature des informations recueillies, de la finalité du traitement de données, des bénéfices et risques potentiels liés à sa participation, et du droit d'accès et de rectification des données auprès du médecin.

Cette information apparaît clairement et lisiblement sur un formulaire d'information et de consentement. L'investigateur remettra au patient une copie de ce formulaire d'information et de de consentement éclairé. Chaque patient aura l'opportunité de poser toutes les questions qu'il désire et sera informé de son droit lui permettant de retirer son consentement à tout moment de l'étude sans avoir à en fournir la raison et sans conséquences sur les soins ultérieurs.

A la suite de cette discussion informative, l'investigateur proposera au patient de dater et de signer le formulaire de consentement. Le patient ne pourra être inclus dans l'étude par l'investigateur qu'après avoir obtenu son consentement informé, volontaire et écrit.

Toute modification du formulaire d'information et de consentement destiné au patient devra être soumis à l'approbation du CPP avant son utilisation.

Un exemplaire du formulaire de consentement daté et signé devra être remis au patient. L'investigateur conservera l'original du formulaire de consentement daté et signé dans son dossier d'étude. L'investigateur précisera sur chaque cahier d'observation qu'il a informé le patient de l'étude et qu'il a obtenu son consentement volontaire et par écrit.

14.4 CONFIDENTIALITE

Tous les documents de l'étude remis par le promoteur à l'investigateur et à son personnel désigné sont soumis à une obligation de confidentialité. Leur contenu ne doit en aucun cas

être divulgué à un tiers ne participant pas directement à l'étude, sans l'autorisation écrite préalable du Promoteur.

L'investigateur doit veiller à ce que l'anonymat du patient soit préservé. Un unique code d'identification sera associé à chaque patient et utilisé dans toutes les communications.

14.5 Arret de l'etude

Sur décision de l'Investigateur Principal de cette étude, l'étude pourra s'interrompre à tout moment pour raison médicale. En outre, le Promoteur se réserve le droit d'arrêter l'étude à tout moment si celle-ci ne peut être conduite en accord avec le protocole.

En cas d'arrêt prématuré ou de suspension de l'étude, le responsable de l'étude en informera rapidement l'investigateur et les autorités. Tout le matériel ayant trait à l'étude devra être retourné, détruit ou conservé, selon les directives du promoteur.

14.6 Archivage

Les documents de l'étude doivent être archivés par le centre participant dans un espace dédié et contrôlé d'accès pendant une période de 15 ans, permettant d'assurer la confidentialité et la protection des données à caractère personnel recueillies durant l'étude. Les procédures standards du centre investigateur en matière d'archivage seront appliquées.

14.7 Assurance et financement

Le Promoteur s'engage à souscrire un contrat d'assurance civil pour toute la durée de l'étude, en accord avec la législation des études chez l'homme.

En cas de dommages ou préjudices causés aux sujets et imputables au traitement à l'étude ou à la participation à l'étude, selon la loi et les GCP, MEDTRUM a souscrit une police d'assurance N° 0100534514058 190120 auprès de HDI GLOBAL SE (annexe 1).

Cette police d'assurance couvre la responsabilité du promoteur, de l'investigateur et de toute autre personne impliquée dans l'étude conformément à la loi.

La demande de réparation pouvant suivre d'autres voies que celle de la loi du 20.12.1988, il est recommandé aux investigateurs d'être titulaires d'un contrat d'assurance responsabilité civile pour leurs activités de recherche.

Le financement de l'étude est intégralement assuré par MEDTRUM.

15 DOCUMENTATION ET UTILISATION DES RESULTATS DE L'ETUDE

Toutes les informations relatives à cette étude et non encore publiées, sont confidentielles et demeurent la seule propriété du Promoteur. Le médecin s'engage à n'utiliser ces informations que pour la conduite de l'étude et pour aucun autre motif sauf accord préalable écrit du promoteur à l'exception de communications éventuelles destinées aux représentants des autorités de santé compétentes.

Un rapport de l'étude sera rédigé en fin d'étude et sera soumis à relecture et à l'approbation du Comité Scientifique.

L'ensemble des données de l'étude et des résultats est la propriété exclusive du Promoteur.

Des communications lors de réunions scientifiques et publications dans des journaux scientifiques à comité de lecture seront réalisées sous couvert du Comité scientifique qui validera leur forme et leur contenu en accord avec le Promoteur.

16 CALENDRIER DE L'ETUDE

Le calendrier prévisionnel de l'étude est le suivant :

- Soumissions réglementaires : Octobre 2019
- Obtention des autorisations réglementaires : décembre 2019
- Mise en place de l'étude : janvier à octobre 2020
- Inclusion des patients : janvier 2020 à novembre 2020
- Fin du suivi du dernier patient : mars 2021
- Gel de base de données : juin 2021
- Rapport complet d'étude avec le suivi : décembre 2021

La durée totale de l'étude ou celle de la période de recrutement est cependant susceptible de varier en fonction des délais réglementaires et des capacités de recrutement.

17 REFERENCES BIBLIOGRAPHIQUES

1. Avis de la CNEDiMTS du 30 mai 2017. Pompes à insuline externes, portables et programmables.
2. Hanaire H, Lassmann-Vague V, Jeandidier N, et al. *Treatment of diabetes mellitus using an external insulin pump : the state of the art.* Diabetes Metab 2008;34:401-23
3. LassmannVague V, Clavel S, Guerci B, Hanaire H, Leroy R, Loeuille GA, Mantovani I, Pi nget M, Renard E, Tubiana-Rufi N; Société francophone du diabète (ex ALFEDIAM) Consensus d'expert: *When to treat a diabetic patient using an external insulin pump. Expert consensus. Société Francophone du Diabète* 2009. *Diabetes Metab*. 2010 Feb;36(1):79-85. Epub 2010 Jan 13.
4. Borot S, Benhamou PY, Atlan C, Bismuth E, Bonnemaison E, Catargi B, Charpentier G, Farret A, Filhol N, Franc S, Gouet D, Guerci B, Guilhem I, Guillot C, Jeandidier N, Joubert M, Melki V, Merlen E, Penfornis A, Picard S, Renard E, Reznik Y, Riveline JP, Rudoni S, Schaepelynck P, Sola-Gazagnes A, Tubiana-Rufi N, Verier-Mine O, Hanaire H; Société francophone du diabète (SFD), Société française d'endocrinologie (SFE); Évaluation dans le diabète des implants actifs Group (EVADIAC). *Practical implementation, education and interpretation guidelines for continuous glucose monitoring: A French position statement*. Diabetes Metab. 2018 ;44 :61-72.
5. The Diabetes control and complications trial research group. *The effect of intensive treatment of diabetes on the development and progression of long-term complications in insulin-dependent diabetes mellitus*. N Engl J Med 1993;329:977-86.
6. Misso ML, Egberts KJ, Page M, O'Connor D, Shaw J. *Continuous subcutaneous insulin infusion (CSII) versus multiple insulin injections for type 1 diabetes mellitus*. *Cochrane Database Syst Rev*. 2010;(1):CD005103.
7. Pickup JC, Sutton AJ. *Severe hypoglycaemia and glycaemic control in type 1 diabetes: meta-analysis of multiple daily insulin injections compared with continuous subcutaneous insulin infusion*. *Diabet Med*. 2008;25(7):765-774.
8. Sämann A, Mühlhauser I, Bender R, Hunger-Dathe W, Kloos C, Müller UA. *Flexible intensive insulin therapy in adults with type 1 diabetes and high risk for severe hypoglycemia and diabetic ketoacidosis*. *Diabetes Care*. 2006;29(10):2196-2199.
9. Karges B, Schwandt A, Heidtmann R et al. *Association of insulin pump therapy vs insulin injection therapy with severe hypoglycemia and and Glycemic Control diabetic ketoacidosis among children, adolescents, and young adults with type 1 diabetes*. JAMA. 2017;318(14):1358-66.
10. American Diabetes Association. *Diabetes care in the hospital, nursing home, and skilled nursing facility*. Sec. 13. In Standards of Medical Care in Diabetes – 2015. Diabetes Care 2015 ;38(Suppl. 1) : S80-S85.
11. Leiter LA, et al. *Assessment of the impact of fear of hypoglycemic episodes on glycemic and hypoglycemia management*. Can J Diabetes 2005; 29: 186-92.

12. Zisser H.C, Howard C, Bevier W, and Jovanovičx L. Siphon. *Effects on continuous subcutaneous insulin infusion pump delivery performance*. Journal of Diabetes Science and Technology, Vol. 4, 2010; 98-103.
13. Bally L, Thabit H, Kojzar H, Mader J.K, Qerilmi-Hyseni J, Hartnell S, Tauschmann M, Allen J.M, Wilinska M.E, Pieber T.R, Evans M.L, Hovorka R. *Day-and-night glycaemic control with closed-loop insulin delivery versus conventional insulin pump therapy in free-living adults with well controlled type 1 diabetes: an open-label, randomised, crossover study*. Lancet Diabetes Endocrinol 2017; 5: 261–70
14. *When to treat a diabetic patient using an external insulin pump*. Expert consensus. Société francophone du diabète (ex ALFEDIAM) 2009
15. S.Borot, , S.Franc, Justine Cristant, A.Penfornis, PY.Benhamou, B. Guerci, H. Hanaire, E. Renard, Y. Reznik, C. Simon, G. Charpentier- *Accuracy of a New Patch Pump Based on a Microelectromechanical System (MEMS) Compared to Other Commercially Available Insulin Pumps: Results of the First In Vitro and In Vivo Studies*. Journal of Diabetes Science and Technology 2014, Vol. 8(6) 1133 –1141
16. https://solidaritessante.gouv.fr/IMG/pdf/Rapport_d_activite_du_CEPS_en_2013_version_francaise_.pdf
17. Nathan D.M, Kuenen J, Borg R, Zheng H, Schoenfeld D, Heine R.J for the A1c-Derived Average Glucose (ADAG) Study Group. *Translating the A1c Assay Into Estimated Average Glucose Values. Diabetes care*, volume 31, number 8, august 2008.
18. Lebenthal Y, Lazar L, Benzaquen H, Shlomit S, Philipp M. *Patient perceptions of using the OmniPOD system compared with conventional insulin pumps in young adults with type 1 diabetes*. Diabetes Tech & Therap. 2012;14(5):11-7.
19. Lane WS, Weinrib SL, Rappaport JM, Przestrelski T. *A prospective trial of U500 insulin delivered by OmniPOD in patients with type 2 diabetes mellitus and severe insulin resistance.* Endocr Pract. 2010; 16 : 778-784.
20. Peyrot M, Rubin R.R. *Validity and Reliability of an Instrument for Assessing Health-Related Quality of Life and Treatment Preferences*. Diabetes Care, Volume 28, number 1, January 2005.
21. Bromba M, Campbell F, and Levy B.L. *The Insulin Treatment Satisfaction Questionnaire and Assessment of Satisfaction with a Latest-generation Insulin Pump. Eur Endocrinol.* 2015 Aug; 11(2): 67–69.
22. Bohannon N, Bergenstal R, Cuddihy R, Kruger D, List S, Massaro E, Molitch M, Raskin P, Remtema H, Strowig S, Whitehouse F, Brunelle R.L, Dreon D and Tan M. *Comparison of a Novel Insulin Bolus-Patch with Pen/Syringe Injection to Deliver Mealtime Insulin for Efficacy, Preference, and Quality of Life in Adults with Diabetes: A Randomized, Crossover, Multicenter Study.* Diabetes Technology & Therapeutics, Volume 13, Number 10, 2011.
23. Anderson RT, Skovlund SE, Marrero D, et al. *Development and validation of the Insulin Treatment Satisfaction Questionnaire*, Clin Ther, 2004;26:565–78.

24. Barnard K, Bromba M, de Lange M, et al., *High reported treatment satisfaction in people with type I diabetes switching to latest generation insulin pump regardless of previous therapy*, J Diabetes Sci Technol, 2015;9:231–6

25. Agiostratidou G, Anhalt H, Ball D, Blonde L, Gourgari E, Harriman K.N, Kowalski A.J, Madden P, McElwee-Malloy M, Peters A, Raman S, Reifschneider K, Rubin K and Weinzimer S.A. *Standardizing Clinically Meaningful outcome Measures Beyond HbA1c for Type 1 Diabetes: A Consensus Report of the American Association of Clinical Endocrinologists, the American Association of Diabetes Educators, the American Diabetes Association, the Endocrine Society*, JDRF International, The Leona M. and Harry B. Helmsley Charitable Trust, the Pediatric Endocrine Society, and the T1D Exchange. Diabetes care, volume 40, December 2017, 1622-1630.

26. Battelino T, Danne T, Bergenstal R , Amiel S, Beck R, Biester T, Bosi E, Buckingham B, Cefalu W, Close K, Cobelli C, Dassau E,DeVries JH, Donaghue K, Dovc K,1 Doyle F, Garg S, Grunberger G, Helle Sr, Heinemann L, B. Hirsch, Hovorka R, Jia W, Kordonouri O, KovatchevB, Kowalski A, Laffel L, Levine B, Mayorov A, Mathieu C, Murphy H.R, Nimri R, Nørgaard K, Parkin CG, Renard E, Rodbard D, Saboo B , Schatz D, Stoner K,Urakami T,Weinzimer S, Phillip M : *Clinical Targets for Continuous Glucose Monitoring Data Interpretation: Recommendations From the International Consensus on Time in Range- Diabetes Care Publish Ahead of Print, published online June 8, 2019*

27. Guidance for Industry Diabetes Mellitus — Evaluating Cardiovascular Risk in New Antidiabetic Therapies to Treat Type 2 Diabetes. FDA U.S. Department of Health and Human Services Food and Drug Administration Center for Drug Evaluation and Research (CDER), December 2008.

18 ANNEXES

18.1 ASSURANCE DE L'ETUDE

HDI Global SE
Tour Opus 12 – Défense 9
77 Esplanade du Général de Gaulle
92914 PARIS LA DEFENSE CEDEX
478 913 882 RCS Nanterre
N° SIRET : 478 913 882 000 54

ATTESTATION D'ASSURANCE
RESPONSABILITE CIVILE PROMOTEUR DE RECHERCHES IMPLIQUANT LA PERSONNE HUMAINE

CONTRAT N°

Nous, soussignés **HDI GLOBAL SE** - Direction pour la France - TOUR OPUS 12, 77, Esplanade de la Défense 92914 PARIS LA DEFENSE agissant en qualité d'assureur, attestons par la présente que :

a souscrit un contrat de Responsabilité Promoteur de recherche impliquant la personne humaine sous le numéro ci-dessus référencé.
Ce contrat est conforme aux dispositions légales et réglementaires Françaises sur les recherches impliquant la personne humaine et notamment aux dispositions de la loi 88.1138 du 20/12/1988, modifiée par les textes subséquents notamment la Loi n°2012-300 du 5 Mars 2012 et son décret d'application n°2016-1537 du 16 Novembre 2016, pour la recherche dénommée ci-après :

Nom du promoteur :

Numéro d'enregistrement :
(EUDRACT ou n° fourni par l'ANSM)

Titre de la recherche :

Nombre de patients :

Début et fin prévisionnels :

La garantie est conforme à l'obligation d'assurance instituée par les textes de la loi précitée, article L 1121-10 du Code de la Santé Publique et articles R 1121-4 à R 1121-9 à la charge du promoteur, tant pour sa responsabilité que pour celle des intervenants.

La présente attestation est valable pour la durée de la recherche concernée et sa présentation vaut présomption de garantie à la charge de l'assureur.

Fait , le

Le Courtier
BIOMEDIC INSURE

L'Assureur
HDI GLOBAL SE

HDI Global SE
RCS Nanterre 478 913 882
TOUR OPUS 12 - LA DEFENSE 9
77, Esplanade du Général de Gaulle
F 92914 PARIS LA DEFENSE CEDEX
Tél. : +33 1 44 05 56 00 – Fax : +33 1 44 05 56 66

HDI Global SE
www.hdi.global

Handelsregister: Registered office Hannover
HR Hannover B 60320
VAT registration ID DE 219828782

Chairman of the Supervisory Board: Herbert K. Haas
Executive Board: Dr. Christian Hinsch (Chairman),
Dr. Joachim ten Eicken, Frank Harting, Dr. Edgar Puls,
Dr. Stefan Sigulla, Jens Wohlthat, Ulrich Wollschläger
