# Supplementary material for: Performance and patients’ satisfaction with the A7+TouchCare insulin patch pump system: A randomized controlled non-inferiority study
Source: PLoS One. 2023 Aug 24;18(8):e0289684. doi: 10.1371/journal.pone.0289684 (PMC10449223; doi:10.1371/journal.pone.0289684)
Supplement: Attachment — Submitted filename: Response to editor and reviewers.docx [file pone.0289684.s005.docx]

***Dear Editor,

The manuscript has been revised.***

***You will find answers to each comment below (italics and bold).***

***Major and minor corrections (responding to comments) have been highlighted in yellow in the revised manuscript (“Revised Manuscript with Track Changes”). We also provide a final clean version (“'Manuscript”).***

***We are confident that the quality of the manuscript is now improved, and we hope our revised version will be considered suitable for publication.***

“The reviewers comment that the discussion section should comment on device dysfunction and other adverse events reported for the pump. In addition, the reviewers have requested further detail and/or clarifications regarding the statistical analyses reported in this study.”

***We made a detailed response for each comment.***

Furthermore, the journal's editorial team has noted that the ethics statement in this article reports that "The study was approved by an independent ethic committee" only. Please provide more details regarding the ethics committee that approved this study.

***The study was approved by the following French ethics committee:***

***CPP VI CHU G. MONTPIED – Administration centrale –***

***58 rue Montalembert***

***BP 69 – 63003 CLERMONT FERRAND cedex 1, FRANCE***

***Tél : +33 4 73 75 10 73 – Fax : +33 4 73 75 10 69***

***mail : cpp-sudest6@chu-clermontferrand.fr***

When submitting your revision, we need you to address these additional requirements.

1. Please ensure that your manuscript meets PLOS ONE's style requirements, including those for file naming. The PLOS ONE style templates can be found at 
<https://journals.plos.org/plosone/s/file?id=wjVg/PLOSOne_formatting_sample_main_body.pdf> and 
<https://journals.plos.org/plosone/s/file?id=ba62/PLOSOne_formatting_sample_title_authors_affiliations.pdf>

***The manuscript and attached files have been revised in accordance with Plos One’s style requirement.***

2. Thank you for stating the following financial disclosure:

YES. The study was funded by Medtrum France (Boulogne Billancourt, France).
 
Please state what role the funders took in the study.  If the funders had no role, please state: "The funders had no role in study design, data collection and analysis, decision to publish, or preparation of the manuscript."

If this statement is not correct, you must amend it as needed.

Please include this amended Role of Funder statement in your cover letter; we will change the online submission form on your behalf.

***Amended statement “Medtrum Technologies Inc. Shanghai supported this study. The funders had no role in study design, data collection and analysis. AP (principal investigator) takes full responsibility for the work, including the decision to submit and publish the manuscript. The findings and conclusions in this study are those of the authors and do not necessarily represent the sponsors' views”***

3. Thank you for stating the following in the Competing Interests section:

I have read the journal's policy and the authors of this manuscript have the following competing interests: C.A. declares congress invitations from Eli Lilly, Astrazeneca, and Sanofi; has received speaker honoraria from Diabeloop SA and Eli Lilly;  has served on advisory board panels for Eli Lilly. V.M. has received congress invitations from: Novo-Nordisk, Isis, Sadir, Vitalaire, Orkyn, Medtronic, and speaker fees or consultancy for expertise from: Medtrum, ISIS, Novo-Nordis. J.A. has received congress invitations, speaker honoraria, and consultancy fees from Lilly, Sanofi and Medtronic and has served on advisory board panels for Novonordisk, Ypsomed, Medtrum and Glooko. S.C.  has received no congress invitations, speaker honoraria, and consultancy fees over the last 24 months. D.G.  has received congress invitations, speaker honoraria, and consultancy fees from  Eli-Lilly, Novo-Nordisk, Sanofi-Diabète, Abbott, Medtronic, Astra Zeneca. L.C. declares that she has no engagement, fees or honoraria conflicting with her participation to the project. B.C. has received congress invitations, speaker honoraria, and consultancy fees from Medtronic Dexcom and Abbott. P.S.-B. has received conference invitations, honoraria and consultancies from Abbott Diabetes Care, Eli-Lilly, Novo-Nordisk, Roche Diabetes Care and Ypsomed. C.P. has received no congress invitations, speaker honoraria, and consultancy fees over the last 24 months. C.T. has received honoraria for consultancy, speaker for conferences and congress invitations, from Abbott, Janssen, Lilly, Medtronic, Roche Diabetes Care, Sanofi. A.P. has received congress invitations, speaker honoraria, and consultancy fees from Abbott, Diabeloop, Eli Lilly and Company, Insulet, Lifescan, Medtronic, Novo Nordisk, and Sanofi, and has served on advisory board panels for Abbott, Insulet, Medtrum, Novo Nordisk, and Sanofi. **This does not alter our adherence to PLOS ONE policies on sharing data and materials.**

Please confirm that this does not alter your adherence to all PLOS ONE policies on sharing data and materials, by including the following statement: "This does not alter our adherence to PLOS ONE policies on sharing data and materials.” (as detailed online in our guide for authors <http://journals.plos.org/plosone/s/competing-interests>).  If there are restrictions on sharing of data and/or materials, please state these. Please note that we cannot proceed with consideration of your article until this information has been declared.

Please include your updated Competing Interests statement in your cover letter; we will change the online submission form on your behalf.

***The competing interests section has been updated in line with Plos One requirement.***

4. We note that you have indicated that data from this study are available upon request. PLOS only allows data to be available upon request if there are legal or ethical restrictions on sharing data publicly. For information on unacceptable data access restrictions, please see <http://journals.plos.org/plosone/s/data-availability#loc-unacceptable-data-access-restrictions>.

In your revised cover letter, please address the following prompts:

a) If there are ethical or legal restrictions on sharing a de-identified data set, please explain them in detail (e.g., data contain potentially identifying or sensitive patient information) and who has imposed them (e.g., an ethics committee). Please also provide contact information for a data access committee, ethics committee, or other institutional body to which data requests may be sent.

***Ethical and legal restrictions apply to the data as public data-sharing was not planned in the information letter given to and signed by participants at inclusion in the study. Data-sharing would therefore require an amendment of the information given to patients after acceptance by the ethics committee and then participants.***

***Here is the contact access for the ethics committee that approved the study:***

***CPP VI CHU G. MONTPIED – Administration centrale –***

***58 rue Montalembert***

***BP 69 – 63003 CLERMONT FERRAND cedex 1, FRANCE***

***Tél : +33 4 73 75 10 73 – Fax : +33 4 73 75 10 69***

***mail : cpp-sudest6@chu-clermontferrand.fr***

b) If there are no restrictions, please upload the minimal anonymized data set necessary to replicate your study findings as either Supporting Information files or to a stable, public repository and provide us with the relevant URLs, DOIs, or accession numbers. Please see <http://www.bmj.com/content/340/bmj.c181.long> for guidelines on how to de-identify and prepare clinical data for publication. For a list of acceptable repositories, please see <http://journals.plos.org/plosone/s/data-availability#loc-recommended-repositories>.

We will update your Data Availability statement on your behalf to reflect the information you provide.

5. Your ethics statement should only appear in the Methods section of your manuscript. If your ethics statement is written in any section besides the Methods, please delete it from any other section.

***The ethics statement appears only in the Methods section now.***

6. Please include captions for your Supporting Information files at the end of your manuscript, and update any in-text citations to match accordingly. Please see our Supporting Information guidelines for more information: <http://journals.plos.org/plosone/s/supporting-information>.

***The caption for supporting information (supplemental table one) has been listed at the end of the manuscript as required.***

Reviewers' comments:

Reviewer's Responses to Questions

**Comments to the Author**

1. Is the manuscript technically sound, and do the data support the conclusions?

The manuscript must describe a technically sound piece of scientific research with data that supports the conclusions. Experiments must have been conducted rigorously, with appropriate controls, replication, and sample sizes. The conclusions must be drawn appropriately based on the data presented.

Reviewer #1: Yes

Reviewer #2: Partly

2. Has the statistical analysis been performed appropriately and rigorously?

Reviewer #1: Yes

Reviewer #2: Yes

3. Have the authors made all data underlying the findings in their manuscript fully available?

The [PLOS Data policy](http://www.plosone.org/static/policies.action#sharing) requires authors to make all data underlying the findings described in their manuscript fully available without restriction, with rare exception (please refer to the Data Availability Statement in the manuscript PDF file). The data should be provided as part of the manuscript or its supporting information, or deposited to a public repository. For example, in addition to summary statistics, the data points behind means, medians and variance measures should be available. If there are restrictions on publicly sharing data—e.g. participant privacy or use of data from a third party—those must be specified.

Reviewer #1: Yes

Reviewer #2: No

4. Is the manuscript presented in an intelligible fashion and written in standard English?

PLOS ONE does not copyedit accepted manuscripts, so the language in submitted articles must be clear, correct, and unambiguous. Any typographical or grammatical errors should be corrected at revision, so please note any specific errors here.

Reviewer #1: Yes

Reviewer #2: Yes

5. Review Comments to the Author

Please use the space provided to explain your answers to the questions above. You may also include additional comments for the author, including concerns about dual publication, research ethics, or publication ethics. (Please upload your review as an attachment if it exceeds 20,000 characters)

Reviewer #1: The article “Performance and patients’ satisfaction with the A7+TouchCare insulin patch pump system: a randomized controlled non-inferiority study” by Amadou and colleagues describes a clinical study with insulin pumps. The study is well-designed and results are presented in a comprehensive manner. Thank you for this nice contribution. I only have some minor comments:

General: I would recommend using the term “glucose management indicator (GMI)” instead of “estimated A1C” as this is state of the art and the methods section references the article by Bergenstal et al. that introduced the GMI

***This has been corrected in the text.***

Methods: Obviously there was no inclusion criterion that describes a minimum required duration of pump use before study start. According to Table 1 the minimum duration was 0.1 years. This appears too short to be able to differentiate study outcomes from improvements in HbA1c due to change from MDI to CSII.

***We understand your concern, but the participants were randomized at baseline, so the effect of the switch from MDI to CSII is supposed to be the same in the two groups. Most patients had been using a pump for 5-7 months. The patients' motivation was to have the opportunity to test an innovative and smaller device. The aim of the study was not to demonstrate superiority of the Medtrum device but to show that the results were similar to Insulet under comparable conditions.***

Results and Discussion: It seems like there is a kind of safety issue with the A7+TouchCare pumps, because 7 patients dropped out due to intolerance or device dysfunction and in general, there were more adverse events with this pump. I think this is should be paid more attention in the discussion of the article.

***There are indeed more AEs and dysfunctions reported in the A7+ Touchcare group. It appeared after discussion with the centres that patients who had already been using the Omnipod pump for several months did not report all events because they were used to their occurrence. On the other hand, in the A7+ Touchcare group, patients who received minimal and rapid training did not always have a perfect mastery of the devices and probably had a more systematic notification of events. Furthermore, it should be remembered that the study was carried out in the context of the covid epidemic, with support from the health care teams that was somewhat different from a normal context, which may have increased the anxiety of the patients in the A7+ Touchcare group when events occurred.***

Author contributions: All investigators all listed as authors, but most of them did not make any further contributions to the manuscript. This is not conform with the ICMJE authorship criteria; e.g. critically revising the manuscript is another required contribution.

***The manuscript has been sent for review and validation to all co-investigators. The author contribution statement has now been updated.***

Reviewer #2: Here are some comments:

Statistical methods:

“In addition, univariate analyses were performed on age in class (according to
the median value), gender, diabetes mellitus type, center, and duration of diabetes. Variables with a p-value less than 0.05 were included in the multivariate model using a stepwise strategy.” My comment here is that conclusions on these variables should be drawn from a full model i.e. a multiple model including all variables. The stepwise strategy is not needed and if any adjustment for the primary analysis is done, why not adjust for all?

***Indeed, only one variable was significant at the 5% level, so we removed the stepwise strategy in the article since it was considered but not used.***

Table 1. Why include statistical tests. Since this is a randomized trial this is not needed. Read about “the table 1 fallacy” and point 15 in the consort checklist <http://www.consort-statement.org/checklists/view/32--consort-2010/510-baseline-data>.

***You are right, the p-values should not be displayed in table 1. We removed it from the table.***

How was the p-value for non-inferiority calculated (table 2)? Probably through the lsmeans statement, include this in the methods.

***We have added this precision in the text.***

Table S1: Please indicate which test that is used and double check the p-values, is the result for “Redness” correct?

***Thank you for your attention. The value for redness was wrong (but still not significant) and has been modified.***

Why is the “other adverse events” not discussed and is the conclusion “Overall, the A7+TouchCare may be a safe and valuable alternative to Omnipod..” correct?

***Only the main adverse events usually described in the use of pumps and sensors were retained, the other AEs being minor. In addition, it should be noted that there is a potential bias because the patients in the Omnipod pump group had all been using the system for several months prior to inclusion and were therefore already sufficiently tolerant. This had the consequence of reducing or even not reporting tolerance problems that patients in this group are used to. We agree to change the conclusion sentence to “Overall, the A7+TouchCare may be a valuable alternative to Omnipod or other insulin patch pumps in some patients with diabetes.”***

Minor: The following sentence is repeated: “The inclusion of patients already using a pump was done to facilitate recruitment into the study. The inclusion of patients already using a pump was done to facilitate recruitment into the study.”

***This has now been corrected in the text.***

6. PLOS authors have the option to publish the peer review history of their article ([what does this mean?](https://journals.plos.org/plosone/s/editorial-and-peer-review-process#loc-peer-review-history)). If published, this will include your full peer review and any attached files.


If you choose “no”, your identity will remain anonymous but your review may still be made public.


**Do you want your identity to be public for this peer review?** For information about this choice, including consent withdrawal, please see our [Privacy Policy](https://www.plos.org/privacy-policy).

Reviewer #1: No

Reviewer #2: No
