# Supplementary material for: Performance and patients’ satisfaction with the A7+TouchCare insulin patch pump system: A randomized controlled non-inferiority study
Source: PLoS One. 2023 Aug 24;18(8):e0289684. doi: 10.1371/journal.pone.0289684 (PMC10449223; doi:10.1371/journal.pone.0289684)
Supplement: Attachment — Submitted filename: Response to Reviewers.docx [file pone.0289684.s006.docx]

Dear Editor,

The manuscript has been revised.

You will find answers to each comment below (italics and bold).

Major and minor corrections (responding to comments) have been highlighted in yellow in the revised manuscript (“Revised Manuscript with Track Changes”). We also provide a final clean version (“'Manuscript”).

We are confident that the quality of the manuscript is now improved, and we hope our revised version will be considered suitable for publication.

The original comments of reviewers are written in blue and our response in black just below.

This is an interesting and well written study, as we lack RCTs regarding the achievements of insulin patch pumps. The current report adresses both performance and satisfaction with a new insulin patch pump in an appopriate, randomized design.

My main concern is that the abstract does not mention about the flow chart and premature study withdrawals, which were more frequent in the Touchcare group, leading to a desequilibrium between the two groups in the per protocol analysis.

🡪 We have modified the abstract and added the number of premature withdrawals in each group.

Regarding satisfaction, the sentence on page 12 about the preference score is unclear, as the insulin management system, opposed to the patch insulin delivery system and the PDM, have not been clearly defined in the Materials section.

🡪 We agree that the sentence is not clear. The insulin management system designs the patch insulin delivery system and the PDM as a whole. We propose to modify the sentence by writing: “The preference score was 3.7±1.5 for the patch insulin delivery system, 1.9±1.3 for the PDM and 3.2±1.4 for the insulin management system (designing the patch insulin delivery and PDM as a whole).

Minor comment: in the Method section, please state whether the fast insulin analog URLI was allowed or not.

🡪 The fast insulin analog URLI was not allowed in this study as it was not available in France at the moment of the study protocol development.

Dr Coralie Amadou, for all authors.
